# Publisher Correction: Bistable and photoswitchable states of matter

**DOI:** 10.1038/s41467-018-05789-y

**Published:** 2018-08-07

**Authors:** Brady T. Worrell, Matthew K. McBride, Gayla B. Lyon, Lewis M. Cox, Chen Wang, Sudheendran Mavila, Chern-Hooi Lim, Hannah M. Coley, Charles B. Musgrave, Yifu Ding, Christopher N. Bowman

**Affiliations:** 1 Department of Chemical and Biological Engineering, University of Colorado, Boulder, CO 80309 USA; 2 Applied Chemicals and Materials Division, National Institute of Standards and Technology, Boulder, CO 80305 USA; 3 Department of Mechanical Engineering, University of Colorado, Boulder, CO 80309 USA; 4 Material Science and Engineering Program, University of Colorado, Boulder, CO 80309 USA; 5 BioFrontiers Institute, University of Colorado, Boulder, CO 80309 USA; 6 Department of Restorative Dentistry, University of Colorado, Anschutz Medical Campus, Aurora, CO 80045 USA; 7 Present Address: Formlabs Inc, 35 Medford St. #201, Somerville, MA 02143 USA; 8 Present Address: Department of Chemistry, Colorado State University, Fort Collins, CO 80523 USA

Correction to: *Nature Communications*, 10.1038/s41467-018-05300-7, published online: 18 July 2018

The original version of this Article contained errors in Fig. 3. In Fig. 3a, the word ‘fluid’ in grey was incorrectly given as ‘solid’ in green, below that, ‘solid’ in green was previously ‘fluid’ in grey. Also, the label on the arrow incorrectly read ‘**TMG** (1 mol%) **HABI-Cl** (3 mol%) 455 nm, 1 min’; the correct version reads ‘**TMG** (1 mol%) **HABI-O-*n*-oct** (4 mol%) 455 nm, 4 min’. In the accompanying legend, the word ‘photobase’ was originally incorrectly given as ‘photoacid’. Additionally, in Fig. 3b, the label on the central image was ‘As is: solid’, rather than the correct ‘As is: fluid’. This has been corrected in both the PDF and HTML versions of the Article.

