# Supplementary material for: Bistable and photoswitchable states of matter
Source: Nat Commun. 2018 Jul 18;9:2804. doi: 10.1038/s41467-018-05300-7 (PMC6052001; doi:10.1038/s41467-018-05300-7)

Supplementary Information

Bistable and Photoswitchable States of Matter

Worrell et al.

Supplementary Note 1:

General information

All stress relaxation, creep, glass transition experiments, and some stress/strain experiments were performed on either a RSA-G2 (TA instruments) or a TA Q800 (TA instruments). Other stress/strain experiments were performed on a MTS Exceed Model E42. Nanoindentation was performed on an EITRE Eitre 3 - Obucat, Nano Imprint Lithography (NIL) System. All AFM experiments were performed on a DI 3100 from Bruker. Mechanophotopatterning was performed on a Nikon Eclipse C*i* microscope with a stretching device fixed to the moveable stage. ^{1}H and ^{13}C-NMR spectra were recorded in $CDCl_3$ (internal standard: 7.26 ppm, ^{1}H; 77.0 ppm, ^{13}C) on a Bruker 400 MHz spectrometer. In-situ infrared spectra were recorded using a Perkin Elmer Spectrum 100-FTIR spectrometer. Column chromatography was carried out employing EMD (Merck) Geduran Silica Gel 60 (40-63 μm). Pre-coated Merck F-254 silica gel plates were used for a thin layer analytical chromatography. All chemicals, solvents, and deuterated solvents were purchased from commercial vendors and were used as received without further purification. Any exceptions are noted within the text below and the vendors are noted within the context of use.

Supplementary Methods:

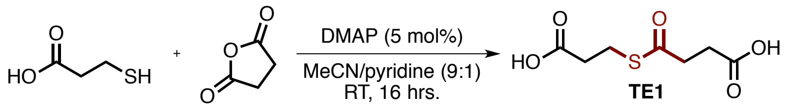

Synthesis of thioester 1* (TE1)**: To a 1.00 L round-bottomed flask equipped with a magnetic stir bar was added 50.0 g (500 mmol, 1.00 equiv) of succinic anhydride which was diluted with 450 mL of anhydrous acetonitrile followed by 50.0 mL of anhydrous pyridine (1.00 M total concentration, 9:1 v/v ratio, MeCN:pyridine) and stirred for ~5 minutes at room temperature to form a homogenous solution. Then, 43.5 mL (53.0 g, 500 mmol, 1.00 equiv) of 3-mercatopropionic acid was added in a single portion followed by 3.05 g (24.98 mmol, 0.05 equiv, 5.00 mol%) of DMAP. The reaction vessel was then sealed with a yellow cap under air and stirred at room temperature overnight (~12 hours). After this period the reaction mixture was concentrated to a thick residue which was dissolved in ~1.00 L of ethyl acetate (EtOAc), acidified with a 1 N aqueous HCl solution (to pH = 1), and the aqueous layer was back-extracted with additional portions of EtOAc (250 mL, 2X); the combined organics were dried over Na_2SO_4, filtered, and concentrated. ***Note: a smaller version of this work-up procedure can be employed to check the conversion of this reaction before final work-up of the larger batch. The white solid obtained after evaporation of the solvent was dissolved into a minimal amount of dichloromethane (DCM, ~100 mL) with rapid stirring using a football shaped magnetic stir bar and mild heating with a heat gun; after complete dissolution, the desired product was precipitated using a large excess of hexanes (~1.00 L) which was added steadily to the stirring mixture. Filtration of the precipitated material and additional washes with smaller portions of hexanes (~250 mL, 2X) yielded 94.6 gram (92% yield) of the title compound (**TE1**) as a white solid which was used in all subsequent studies with no further purifications. ***Note***: this reaction has been successfully scaled up to a 1.00 mole scale (100 g of the succinic anhydride employed) with no changes in the stoichiometry, relative concentrations, reaction times, or work-up, which gave no significant changes in purity or yield of the final product.

TE1: 92% yield; white solid; R_f = n/a; ^{1}H NMR (400 MHz, MeOD-d_3, 25°C): δ= 3.09 (t, J=5.4 Hz, 2H), 2.87 (t, J=5.4 Hz, 2H), 2.63-2.56 (m, 4H); ^{13}C NMR (100 MHz, MeOD-d_3, 25°C) 199.32, 175.57, 175.19, 39.34, 35.08, 29.72, 24.87.

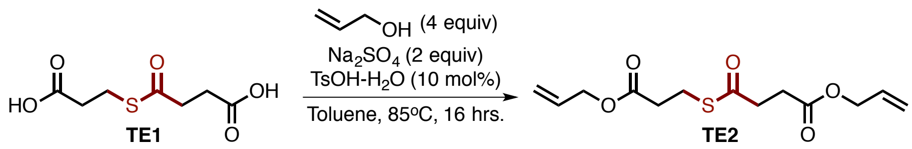

***Synthesis of thioester 2* (TE2)**: To a 250 mL round-bottomed flask equipped with a magnetic stir bar was added 10.0 g (48.5 mmol, 1.00 equiv) of **TE1** (synthesis of which is detailed above), 13.8 gram (97.0 mmol, 2.00 eq) of anhydrous sodium sulfate (Na_2SO_4), 922 mg (4.85 mmol, 0.10 equiv, 10.0 mol%) of *p*-toluenesulfonic acid monohydrate (TsOH-H_2O) and diluted with 100 mL (0.50 M) of reagent grade toluene. To this stirring suspension, 13.2 mL (11.3 g, 194 mmol, 4.00 equiv) of allyl alcohol was added in a single portion via pipette; the flask was equipped with a reflux condenser (open to air), placed into an oil bath, and heated to 85°C with rapid stirring for 12 hours. After this time the reaction mixture was allowed to cool to room temperature and the solids were filtered, the filter cake was washed with additional portions of reagent grade toluene (25 mL, 2X), and concentrated to yield a clear syrupy residue (bath was placed at 60°C to remove any traces of excess allyl alcohol). The crude residue was directly submitted to column chromatography (10% → 20% → 30% EtOAc/hexanes) and concentration of the fractions containing the desired material (R_f = 0.19, TLC conditions: 10% EtOAc/hexanes) yielded 10.9 gram (79% yield) of the title compound (**TE2**) as a clear oil which was found to be sufficiently pure and was used in all subsequent studies with no further purification.

TE2: 82% yield; non-viscous, clear oil; R_f = 0.19 (TLC conditions: 10% EtOAc/hexanes); ^{1}H NMR (400 MHz, $CDCl_3$, 25°C): δ= 5.95 – 5.85 (m, 2H), 5.34 – 5.21 (m, 4H), 4.60 – 4.57 (m, 4H), 3.14 (t, *J*=5.4 Hz, 2H), 2.89 (t, *J*=5.4 Hz, 2H), 2.70 – 2.63 (m, 4H); ^{13}C NMR (100 MHz, $CDCl_3$, 25°C) 197.56, 171.66, 171.39, 132.03, 132.01, 118.66, 118.60, 65.65, 65.61, 38.48, 34.45, 29.21, 24.07.

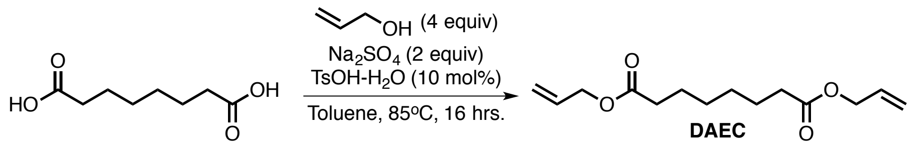

Note: See **Supplementary Figure 11** for use of **DAEC** as the "- thioester" control.

Synthesis of diallylester control (**DAEC**): To a 250 mL round-bottomed flask equipped with a magnetic stir bar was added 10.0 gram (57.4 mmol, 1.00 equiv) of suberic acid, 16.3 gram (115 mmol, 2.00 eq) of anhydrous sodium sulfate (Na_2SO_4), 1.09 gram (5.74 mmol, 0.10 equiv, 10.0 mol%) of *p*-toluenesulfonic acid monohydrate ($TsOH\text{-}H_2O$), and this was diluted with 115 mL (0.50 M) of reagent grade toluene. To this stirring suspension, 15.6 mL (13.3 g, 230 mmol, 4.00 equiv) of allyl alcohol was added in a single portion via pipette; the flask was equipped with a reflux condenser (open to air), placed into an oil bath, and heated to 85°C with rapid stirring for 12 hours. After this time the reaction mixture was allowed to cool to room temperature and the solids were filtered, the filter cake was washed with additional portions of reagent grade toluene (25 mL, 2X), and the filtrate was concentrated to yield a clear syrupy residue (bath was placed at 60°C to remove any traces of excess allyl alcohol). The crude residue was dissolved in EtOAc (~200 mL), transferred to a 500 mL separatory funnel, washed with an aqueous solution of $NaHCO_3$ (~100 mL, 2X), then brine (~100 mL, 1X). The combined organics were dried over Na_2SO_4, filtered, and concentrated to yield 12.6 gram (86% yield) of the title compound (**DAEC**) as a clear oil which was found to be sufficiently pure and was used in all subsequent studies with no further purification. ***Note***: often the material would have a small amount of an unknown precipitate suspended in the oil which could be easily removed by filtering the oil through a 0.2 μm syringe filter.

DAEC: 86% yield; non-viscous, clear oil; R_f = 0.33 (TLC conditions: 10% EtOAc/hexanes, visualized by $KMnO_4$ stain); ^{1}H NMR (400 MHz, $CDCl_3$, 25°C): δ= 5.96 – 5.86 (m, 2H), 5.33 – 5.21 (m, 4H), 4.58 – 4.56 (m, 4H), 2.32 (t, *J*=7.42 Hz, 4H), 1.67 – 1.60 (m, 4H), 1.38 – 1.30 (m, 4H); ^{13}C NMR (100 MHz, $CDCl_3$, 25°C) 132.42, 118.27, 65.10, 34.28, 28.87, 24.86.

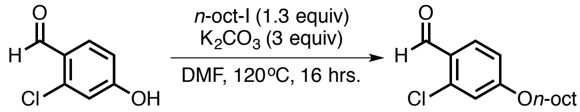

HABI-1: To a 500 mL round-bottomed flask equipped with a magnetic stir bar was added 5.00 gram (32.1 mmol, 1.00 equiv) of 2-chloro-4-hydroxybenzaldehyde which was diluted with 75.0 mL (0.43 M) of reagent grade DMF. To this stirring solution was added 7.52 mL (10.0 gram, 41.7 mmol, 1.30 equiv) of 1-iodooctane, 13.3 gram (96.3 mmol, 3.00 equiv) of potassium carbonate (K_2CO_3), and this suspension was heated to 120°C for 16 hours. After this time the suspension was allowed to room temperature and the solids were filtered, the filter cake was washed with additional small portions of EtOAc (~25.0 mL, 3X), and the filtrate was concentrated to yield an orange residue. The crude residue was dissolved in EtOAc (~200 mL), transferred to a 500 mL separatory funnel, washed with water (~100 mL, 2X), then brine (~100 mL, 1X). The combined organics were dried over Na_2SO_4, filtered, and concentrated to dryness. This crude residue was directly submitted to column chromatography (0% → 5% → 10% EtOAc/hexanes) and the fractions containing the desired compound (R_f = 0.30, TLC conditions: 10% EtOAc/hexanes) were concentrated to yield 7.84 gram (91%) of the title compound as a slightly yellow oil.

HABI-1: 91% yield; slightly yellow oil; R_f = 0.30 (TLC conditions: 5% EtOAc/hexanes); ^{1}H NMR (400 MHz, $CDCl_3$, 25°C): δ= 10.32 (d, *J*=0.82 Hz, 1H), 7.88 (d, *J*=8.73, 1H), 6.92 (d, *J*=2.38, 1H), 6.87 (ddq, *J*=8.7, 2.4, 0.9, 0.8, 1H), 4.02 (t, *J*=6.55, 2H), 1.83 – 1.76 (m, 2H), 1.49 – 1.41 (m, 2H), 1.39 – 1.24 (m, 8H), 0.89 (t, *J*=6.79, 3H); ^{13}C NMR (100 MHz, $CDCl_3$, 25°C): δ= 188.73, 164.40, 139.87, 131.12, 125.93, 115.78, 114.18, 68.98, 31.92, 29.39, 29.33, 29.06, 26.03, 22.79, 14.24.

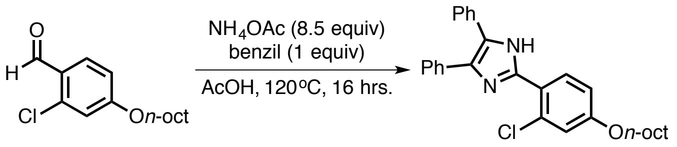

HABI-2: To a 250 mL round-bottomed flask equipped with a magnetic stir bar was added 7.84 gram (29.2 mmol, 1.00 equiv) of **HABI-1**, which was dissolved in 115 mL (0.25 M) of glacial acetic acid. To this clear solution was added 6.14 gram (29.2 mmol, 1.00 equiv) of benzil, followed by 19.1 gram (249 mmol, 8.50 equiv) of ammonium acetate to form a suspension. The flask was equipped with a reflux condenser, placed under a mild vacuum for ~5 minutes, then opened to an atmosphere of argon; this procedure was repeated 3x times. The suspension was then heated to 120ºC, forming a solution at ~100ºC, and allowed to heat at this temperature for 16 hours. After this period the reaction mixture was allowed to cool to room temperature and the volatiles were removed under reduced pressure to give a crude residue. This residue was dissolved in DCM (~200 mL), transferred to a 500 mL separatory funnel, washed with an aqueous solution of $NaHCO_3$ (~100 mL, 2X), and brine (~100 mL, 1X). The combined organics were dried over Na_2SO_4, filtered, and concentrated to give 13.1 gram (98%) yield of the title compound as a yellow/beige solid which was found to be sufficiently pure for our purposes and was utilized directly in the next step with no further purification.

HABI-2: 98% yield; yellow/beige solid; $R_f = 0.30$ (TLC conditions: 10% EtOAc/hexanes); ^{1}H NMR (400 MHz, $CDCl_3$, 25ºC): δ= 10.08 (bs, 1H), 8.34 (d, *J*=8.75 Hz, 1H), 7.67 (bs, 2H), 7.48 (bs, 2H), 7.42 – 7.24 (bm, 6H), 3.99 (t, *J*=6.55, 2H), 1.80 (m, 2H), 1.51 – 1.43 (m, 2H), 1.40 – 1.26 (m, 8H), 0.90 (t, *J*=6.73 Hz, 3H); ^{13}C NMR (100 MHz, $CDCl_3$, 25ºC) 159.87, 143.60, 131.92, 130.45, 129.08, 128.48, 127.88, 127.24, 127.10, 120.69, 116.01, 114.45, 77.36, 77.36, 68.64, 31.95, 29.47, 29.37, 29.24, 26.12, 22.81, 14.26.

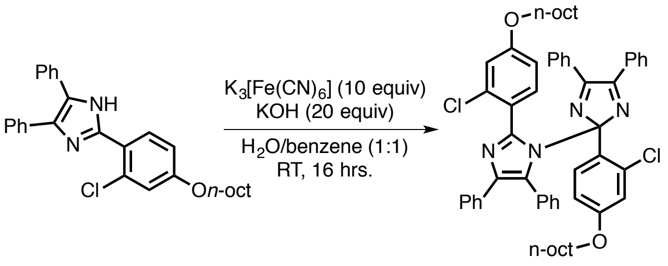

Note: This procedure was performed, to the best of our abilities, in the absence of any UV-light.

HABI-O-n-oct: To a 250 mL round-bottomed flask equipped with a magnetic stir bar and a 60.0 mL addition funnel was added 1.00 gram (2.18 mmol, 1.00 equiv) of **HABI-2** and this was diluted with 31.0 mL (~0.02 M) of reagent grade benzene. To a separate 100 mL round-bottomed flask equipped with a magnetic stir bar was added 2.44 gram (43.6 mmol, 20.0 equiv) of KOH, diluted with 31.0 mL (~0.02 M, total dilution of ~0.04 M) of distilled water, and allowed to stir for ~10 minutes or until the solids had completely dissolved. After this period, 7.18 gram (21.8 mmol, 10.0 equiv) of potassium ferricyanide ($K_3[Fe(CN)_6]$) was added in a single portion and allowed to stir for an additional ~10 minutes or until the solids had completely dissolved. The fully homogenous KOH/$K_3[Fe(CN)_6]$ solution was placed into the 60.0 mL addition funnel, washing with minimal amounts of water to assure complete transfer of the reagents. The entire flask was placed under a mild vacuum and opened to an atmosphere of argon (balloon, 1 atm). This procedure was repeated 3 times, keeping the argon filled balloon equipped to the reaction after the final cycle. The KOH/$K_3[Fe(CN)_6]$ solution was then slowly added dropwise to the reaction with vigorous stirring over the course of 30 minutes. It was noted during this period that the biphasic reaction turned from a light-yellow solution into a bright blue/green biphasic solution upon complete addition of the reagents. The reaction was wrapped in tin foil and allowed to stir vigorously for 16 hours at room temperature. After this period the reaction was transferred to a 250 mL separatory funnel, the aqueous layer was removed and the organics were washed with distilled water (~100 mL, 2X), brine (~100 mL, 1X), dried over Na_2SO_4, filtered, and concentrated to yield a crude yellow/blue foam. The crude residue was directly submitted to column chromatography (5% → 10% EtOAc/hexanes) and concentration of the fractions containing the desired material (R_f = 0.20, TLC conditions: 10% EtOAc/hexanes) yielded 948 gram (95% yield) of the title compound (**HABI-O-*n*-oct**) as a clear oil which was found to be sufficiently pure and was used in all subsequent studies with no further purification.

HABI-O-n-oct: 95% yield; yellow foam; R_f = 0.20 (TLC conditions: 10% EtOAc/hexanes); ^{1}H NMR (400 MHz, $CDCl_3$, 25°C): δ= 7.62 (d, *J*=8.3 Hz, 3H), 7.55 (t, *J*= 7.4 Hz, 1H), 7.43 (dd, *J*= 15.5, 7.4 Hz, 4H), 7.38 – 7.33 (m, 1H), 7.20 – 7.04 (m, 9H), 6.77 – 6.74 (m, 2H), 6.57 (d, *J*= 8.9, 1H), 6.41 (d, *J*= 8.9 Hz, 1H), 6.01 (dd, *J*= 8.9, 2.6 Hz, 1H), 3.83 – 3.78 (m, 2H), 3.73 – 3.61 (m, 2H), 1.69 (p, *J*= 7.1 Hz, 4H), 1.43 – 1.26 (m, 20H), 0.93 – 0.86 (m, 6H); ^{13}C NMR (100 MHz, $CDCl_3$, 25°C) 168.13, 165.05, 159.63, 159.53, 144.16, 138.21, 135.34, 135.25, 134.80, 133.22, 132.31, 132.28, 131.06, 131.00, 130.00, 129.95, 129.67, 129.10, 128.40, 127.86, 127.77, 127.74, 127.61, 127.11, 126.98, 126.88, 126.08, 116.89, 114.55, 113.08, 112.06, 110.80, 68.38, 68.22, 31.98, 31.93, 29.50, 29.42, 29.40, 29.36, 29.21, 29.07, 26.19, 26.02, 22.83, 22.78, 14.27, 14.24.

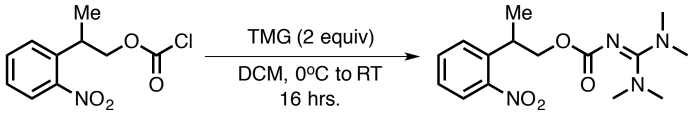

NPPOC-Cl NPPOC-TMG (**PB**)

Synthesis of the TMG-appended photobase (**PB**): To a 100 mL round-bottomed flask equipped with a magnetic stir bar was added 2.00 gram (8.23 mmol, 1.00 equiv) of 2-(2-nitrophenyl)propyl chloroformate (~95% pure, obtained from Sigma-Aldrich) and diluted with 40.0 mL (0.20 M) of reagent grade DCM. This stirring solution was cooled to 0°C by the application of an ice bath and 1.03 mL (946 mg, 8.21 mmol, 2.00 equiv) of 1,1,3,3-tetramethylguanidine (TMG). This clear solution was allowed to stir for 1 hour at 0°C, then the ice bath was removed and the reaction was allowed to stir for 16 hours. After this period the reaction was quenched by the addition of brine (~50.0 mL), transferred to a separatory funnel, washed with additional portions of brine (~50.0 mL, 2X), and the combined organics were dried over Na_2SO_4, filtered, and concentrated to dryness. This crude residue was directly submitted to column chromatography (0% → 1% → 5% MeOH/DCM), and the fractions containing the desired compound (R_f = 0.47, TLC conditions: 10% MeOH/DCM) were concentrated to yield 1.29 gram (98%) of the title compound (**PB**) as a slightly yellow oil. This oil was found to have solidified as a waxy off-white solid after several hours of cooling in a -20°C freezer. Alternatively, it was found that seeding this viscous oil with a small quantity of a previously crystallized batch of NPPOC-TMG also greatly accelerated the speed of solidification.

PB: 98% yield; waxy off-white solid; R_f = 0.47 (TLC conditions: 10% MeOH/DCM); ^{1}H NMR (400 MHz, $CDCl_3$, 25°C): δ= 7.76 – 7.73 (m, 1H), 7.57 – 7.52 (m, 2H), 7.35 – 7.31 (m, 1H), 4.29 (d, *J*= 7.16 Hz, 2H), 3.75 – 3.68 (m, 1H), 2.82 (s, 12H), 1.38 (d, *J*= 6.94 Hz, 3H); ^{13}C NMR (100 MHz, $CDCl_3$, 25°C) 166.29, 160.29, 150.45, 138.49, 132.64, 128.58, 127.08, 68.49, 39.85, 34.01, 18.72.

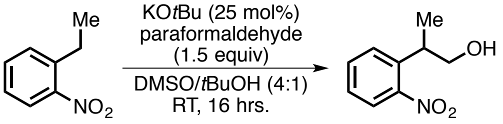

Synthesis of the photoacid precursor **(PA-OH)**: To a 100 mL round-bottomed flask equipped with a magnetic stir bar was added 5.00 gram (33.1 mmol, 1.00 equiv) of 1-ethyl-2-nitrobenzene using a Pasteur pipette and this was diluted with 30 mL of reagent grade DMSO. To a separate 20 mL scintillation vial equipped with a magnetic stir bar was added 1.49 gram (49.7 mmol, 1.50 equiv) of paraformaldehyde followed by 929 mg (8.27 mmol, 0.25 equiv, 25.0 mol%) of potassium *tert*-butoxide (KO*t*Bu) and these were suspended in 9.00 mL of *tert*-butanol (*t*-BuOH). To this stirring suspension, 6.00 mL of reagent grade DMSO was added and the suspension formed a not fully clear but manageable (easily transferrable) solution. This solution was pipetted into the stirring DMSO solution containing 1-ethyl-2-nitrobenzene (45 mL total, total concentration 0.75 M, 4:1 DMSO/*t*-BuOH) and the reaction mixture was stirred at room temperature for 16 hours. After this period the reaction was diluted with water (~50 mL), transferred to a separatory funnel, and extracted with EtOAc (~100 mL, 2X). The combined organic layer was washed with water (~100 mL, 2X), then brine (~100 mL, 1X), dried over Na_2SO_4, filtered, and concentrated to dryness. This crude residue was directly submitted to column chromatography (10% → 20% → 30% EtOAc/hexanes) and the fractions containing the desired compound (R_f = 0.25, TLC conditions: 30% EtOAc/hexanes) were concentrated to yield 5.66 gram (94%) of the title compound (**PA-OH**) as a yellow/orange oil which was found to be sufficiently pure and was used in all subsequent studies with no further purifications.

PA-OH: 94% yield; yellow/orange oil; R_f = 0.25 (TLC conditions: 30% EtOAc/hexanes); ^{1}H NMR (400 MHz, $CDCl_3$, 25°C): δ= 7.77 (dd, *J*= 8.14, 1.37 Hz, 1H), 7.62 – 7.58 (m, 1H), 7.52 (dd, *J*= 7.94, 1.49 Hz, 1H), 7.38 (ddd, *J*= 7.27, 1.49 Hz, 1H), 3.86 – 3.76 (m, 2H), 3.58 – 3.50 (m, 1H), 1.70 (bs, 1H), 1.35 (d, *J*= 6.92 Hz, 3H); ^{13}C NMR (100 MHz, $CDCl_3$, 25°C) 150.83, 138.21, 132.79, 128.31, 127.33, 124.24, 68.01, 36.49, 17.69.

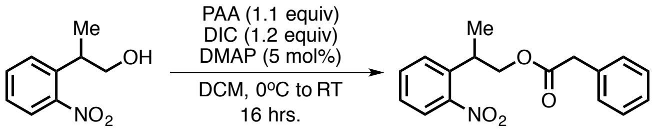

Synthesis of the photoacid **(PA)**: To a 100 mL round-bottomed flask equipped with a magnetic stir bar was added 1.00 gram (5.52 mmol, 1.00 equiv) of **PA-OH** and this was diluted with 20.0 mL (0.30 M) of reagent grade DCM. To this was added 902 mg (6.63 mmol, 1.20 equiv) of phenylacetic acid, 34.0 mg (0.28 mmol, 0.05 equiv, 5.00 mol%) of DMAP, and this suspension was allowed to stir for ~10 minutes until it became a solution. The reaction was then added 1.04 mL (836 mg, 6.62 mmol, 1.20 equiv) of diisopropylcarbodiimide (DIC) was added dropwise; the reaction mixture was allowed to slowly warm to room temperature and stir for 16 hours. After this period a fine precipitate had formed which was vacuum filtered through a filter paper-topped Büchner funnel, washed with additional small portions of DCM (~10.0 mL, 2X), and the combined filtrate was concentrated to give a cloudy thick residue. This residue was dissolved in a minimal amount of EtOAc (~20.0 mL) and, again, filtered through a filter paper-topped Büchner funnel. The combined filtrate was concentrated to give an almost clear residue which was directly submitted to column chromatography (5% → 10% EtOAc/hexanes) and the fractions containing the title compound (R_f = 0.22, TLC conditions: 10% EtOAc/hexanes) were concentrated to give 1.64 gram (99%) of the title compound (**PA**) as a slightly yellow oil which was found to be sufficiently pure and was used in all subsequent studies with no further purifications.

PA: 99% yield; slightly yellow oil; R_f = 0.22 (TLC conditions: 10% EtOAc/hexanes); ^{1}H NMR (400 MHz, $CDCl_3$, 25°C): δ= 7.74 (ddd, *J*=8.1, 1.4, 0.4 Hz, 1H), 7.52 – 7.45 (m, 1H), 7.37 – 7.22 (m, 5H), 7.19 – 7.15 (m, 2H), 4.28 – 4.20 (m, 2H), 3.70 (h, *J*=7.0 Hz, 1H), 3.55 (d, *J*=2.9 Hz, 2H), 1.30 (d, *J*=6.98 Hz, 3H); ^{13}C NMR (100 MHz, $CDCl_3$, 25°C) δ= 171.37, 150.50, 137.30, 133.92, 132.70, 129.37, 128.66, 128.30, 127.50, 127.18, 124.55, 68.60, 41.44, 33.15, 17.78.

Stress relaxation, creep, and stress/strain experiments:
All stress relaxation and creep experiments were performed in tensile elongation using a Q800 DMA (TA Instruments) or a RSA-G2 (TA Instruments). For stress relaxation, the built-in stress relaxation mode was used, with a 10% strain for ambient temperature tests. For temperature stepping tests, which are performed repeatedly on the same sample, a 4% strain was used to avoid significant sample deformation between scans. For stress/strain experiments a strain rate of 0.25 mm/min was used. Films were cut into rectangular sections and measured with calipers prior to loading into the DMA.

Dynamic mechanical analysis of the glass transition temperature:
The glass transition temperature was determined on a Q800 DMA (TA Instruments) using a ramp rate of 3°C/min and a frequency of 3 Hz, with a fixed oscillatory strain of 0.025%. Films were cut into rectangular sections and measured with calipers prior to loading into the DMA.

MTS – stress/strain experiments:
Some stress/strain experiments were performed on an MTS Exceed Model E42 using the built in software. This instrument was only utilized to obtain the data shown below, wherein a strain rate of either 0.75 mm/min or 0.10 mm/min was used.

Rheometry:
Frequency sweeps were performed on an ARES rheometer (TA instruments) with a temperature controlled stage installed, which served as the bottom plate under the sample. For the top plate, an 8 mm diameter quartz plate was used, with a mirror on the top fixture that directed light from a light guide through the sample. Quartz was employed rather than a standard metal plate to allow for irradiation of the sample to polymerize *in situ*, as well as to minimize temperature gradients through the sample. The polymerization was initiated using a mercury arc lamp (Acticure, EXFO) using a 365 nm filter and a light intensity of 3 mW/cm^2. During irradiation, the storage and loss moduli were monitored, using a constant strain of 5% and frequency of 5 rad/s. The time scan was stopped and lamp turned off once the modulus had reached a plateau value. Once the polymerization was completed, the temperature was equilibrated to the desired value and frequency scans were performed using 1-5% strain over several orders of magnitude of frequency.

Computational methods:
Calculations were performed with Gaussian 09 computational chemistry package, using Trestles Supercomputer, XSEDE. Stationary geometries (reactants, transition states and products) were computed for all systems studied using density functional theory based on the M06 density functional and 6-31+G** basis set. The M06 functional was chosen because it has been parameterized with experimental thermodynamic data, should provide a reliable description of the molecular structures for the reactions of interest. An adequate treatment of solvent is crucial to correctly describe reactions involving a polar TS, such as those involving nucleophilic attacks, which are of particular interest here. Therefore, the implicit polarized continuum solvation model (CPCM) was employed in all calculations to treat the solute-solvent electrostatic interactions. The modeled solvent was chosen as ethyl acetate to approximate the monomer/polymer environment containing ester functionality. Vibrational force constants were calculated at the M06/6-31+G** level of theory to: 1) verify that the reactant and product structures have only positive vibrational modes, 2) confirm that each TS has only one imaginary mode and that it connects the desired reactant and product structures via Intrinsic Reaction Coordinate (IRC) calculations, and 3) compute entropies, zero-point energies (ZPE) and thermal corrections included in the reported free energies at 298K.

Nanoindentation:

Nanoindentation (as can be seen in Figure 4A) was performed on a EITRE Eitre 3 – Obducat, Nano Imprint Lithography (NIL) System. The nanoindenter is equipped with a lamp (300-400, 20 mW/cm^2), which can irradiate the sample from the top while being compressed from the bottom. A nanopatterned silicon stamp (LightSmyth, SNS-C12-2525-200-P, period - 833.3 nm, grove depth – 200 nm, line width – 416 nm) was used as the standard stamp under all circumstances.

Atomic-Force Microscopy (AFM):

All AFM experiments were performed on a DI 3100 from Bruker, and plots were formed using the preinstalled software.

Mechanophotopatterning:

Mechanophotopatterning (as can be seen in Figure 4B) was performed on a Nikon Eclipse C*i* microscope with a stretching device fixed to the moveable stage. The sample (6×15×0.25 mm) was fixed to the stretching device using clear Scotch tape with a gap of approximately 8 mm and stretched approximately 2 mm (until the birefringence became clear through cross polarizers). The stretched device was set at a 45° angle to the cross polarizers. The buffalo was irradiated through a dynamic light projector (Mightex Polygon 400) integrated into the microscope. The intensity of 400 nm centered LED light was measured at 125 mW/cm^2.

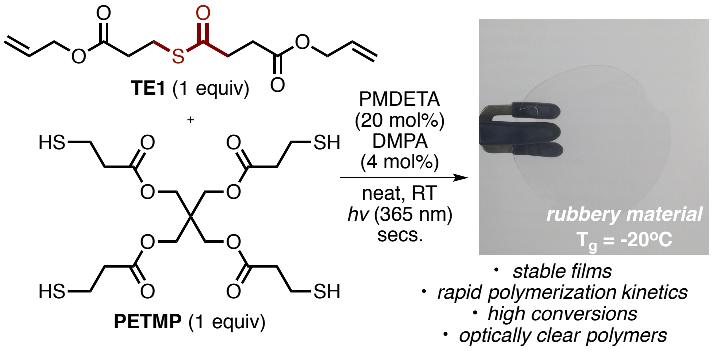

Supplementary Figure 1: A representative procedure for the preparation of a thioester containing network polymer via the photoinitiated thiol-ene reaction (formulation employed in Figure 1C & 1D).

To a 10.0 mL speed mixer vial was added 250 mg (0.87 mmol, 1.00 equiv) of **TE2**, 427 mg (0.87 mmol, 1.00 equiv, "100% excess thiol") of pentaerythritol tetra(3-mercaptopropionate) (PETMP), and 36.4 μL (30.2 mg, 0.17 mmol, 0.20 equiv, 20.0 mol%) of *N,N,N',N'',N''*-pentamethyldiethylenetriamine (PMDETA) each via Pasteur pipettes. This clear resin was then manually mixed with a pipette tip for ~2 minutes to make a homogenous mixture. Following this, approximately 8.91 mg ($3.48x10^{-2}$ mmol, 0.04 equiv, 4.00 mol%) of 2,2-dimethoxy-2-phenylacetophenone (DMPA), which had been crushed with the flat side of a spatula to form a fine powder, was added and the resin was further manually mixed with a pipette tip for an additional ~2 minutes to form a homogeneous mixture. At this time the clear resin was poured between two glass slides treated with Rain-X (ITW Global Brands, Houston, TX) using 250 μm thick spacers (Small Parts Inc., Logansport, IN). The material was irradiated (365 nm, 5.00 $\mu W/cm^2$, room temperature) for ~10 minutes to yield the thiol excess, thioester-containing network polymer. The conversion was found to be essentially quantitative by *in situ* IR, revealing complete consumption of the "ene" species (**S3**). Moreover, it was shown that, due to the quantitative nature of the thiol-ene reaction, any excess of either reactant (ene or thiol) remained unreacted in the final network polymer (**S4**). As pictured above, this procedure generally formed circular samples which were cut utilizing a single edge straight razor blade to form the required rectangular samples. **Note**: this represented the **+thioester +base +free SH** formulation utilized in **S5** (grey line).

+thioester +base ***–free SH*** formulation: The representative procedure outlined above was modified to a 2:1 ene:thiol ratio (as opposed to 1:2 ene:thiol) to ensure that all free thiol was consumed and a similar crosslinking density was preserved (**S5**, blue line)

-thioester **+base +free SH** formulation: The representative procedure outlined above was utilized, however, **DAEC**, which contained no thioester linkage, was employed in the stead of **TE2**; relative stoichiometry of ene:thiol (1:2) was maintained (**S5**, red line).

+thioester ***–base*** **+free SH** formulation: The representative procedure outlined above was utilized, however, PMDETA was not added to the resin (**S5**, green line)

Supplementary Figure 2: Formation of a plano-convex lens from recycled polymer (as detailed in Figure 1D).

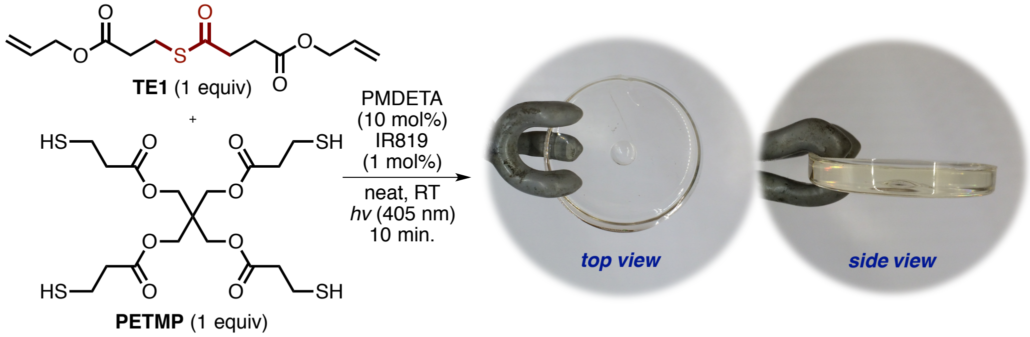

To a 20.0 mL speed mixer vial was added 4.00 gram (13.9 mmol, 1.00 equiv) of **TE2**, 6.82 gram (13.9 mmol, 1.00 equiv) of PETMP, 292 μLs (243 mg, 1.39 mmol, 0.10 equiv, 10.0 mol%) of PMDETA, and 58.0 mg (0.14 mmol, 0.01 equiv, 1.00 mol%) of IR819. This thick residue was manually mixed with a pipette tip until all of the solids (IR819) had dissolved and the clear, slightly yellow residue was loaded into a 16 mL syringe and pushed through a syringe filter (45.0 μm) into a 25.0 mL petri dish. This residue was allowed to settle at room temperature for approximately 1 hour. After this period the dish was placed into a shallow ice bath and irradiated (405 nm, 50.0 mW/cm^2, room temperature) for 10 minutes. The now polymerized sample was allowed to reach room temperature and was removed from the petri dish.

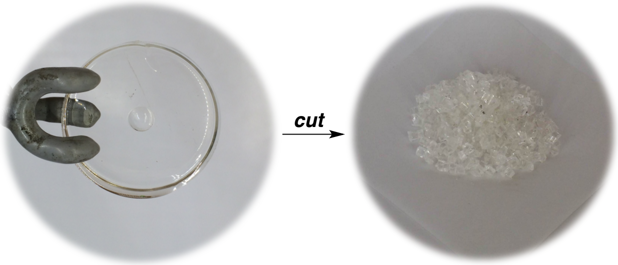

The polymerized sample was cut into length-wise strips and then further cut into small cubes to form our "cut" material. This was done using a single edge straight razor blade. A small amount of this cut sample was utilized to show that the thiol-ene polymerization reaction had reached full conversion by FT-IR.

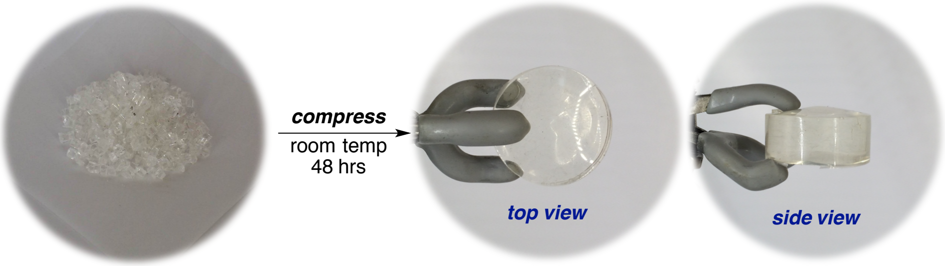

A 60.0 mL plastic syringe, which had been soaked in isopropanol and sonicated for ~1 hour, was loaded with the polymeric cut material. The plunger was pressed down firmly to contact the cut material and several rubber bands were wrapped around both ends of the syringe to further compress the material. This syringe was left at room temperature for 48 hours (2 days) to form the puck of healed material. To remove the puck, the syringe was cut down the side and the puck was easily removed.

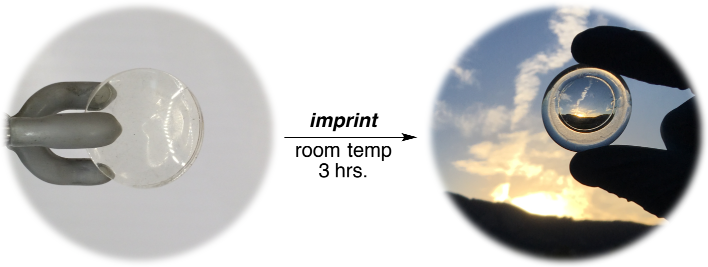

The healed material was placed on top of an optically flat surface (Optosigma BK-7, $\lambda/10$) and then a UV-fused silica plano-convex lens (Thorlabs, Ø1/2", f = 4.0 mm) was placed on top of the material followed by a lens cleaning tissue paper (Thorlabs). This sandwich of material was compressed for three hours under mild pressure at room temperature. Upon removal of the lens from the material a plano-concave lens was formed which, if not further imprinted upon, was indefinitely stable.

A representative procedure for the preparation of a thioester containing network polymers with different catalysts:

To a 10.0 mL speed mixer vial was added 250 mg (0.87 mmol, 1.00 equiv) of **TE2**, 427 mg (0.87 mmol, 1.00 equiv, "100% excess thiol") of pentaerythritol tetra(3-mercaptopropionate) (PETMP), and varying basic catalyst (0.03 mmol, 0.03 equiv, 3.00 mol%) each via Pasteur pipettes. This clear resin was then manually mixed with a pipette tip for ~2 minutes to make a homogenous mixture. Following this, approximately 8.91 mg (3.48×10^{-2} mmol, 0.02 equiv, 2.00 mol%) of 2,2-dimethoxy-2-phenylacetophenone (DMPA), which had been crushed with the flat side of a spatula to form a fine powder, was added and the resin was further manually mixed with a pipette tip for an additional ~2 minutes to form a homogeneous mixture. At this time the clear resin was poured between two glass slides treated with Rain-X (ITW Global Brands, Houston, TX) using 250 μm thick spacers (Small Parts Inc., Logansport, IN). The material was irradiated (365 nm, 5.00 $\mu W/cm^2$, room temperature) for ~10 minutes to give the thiol excess thioester containing network polymer.

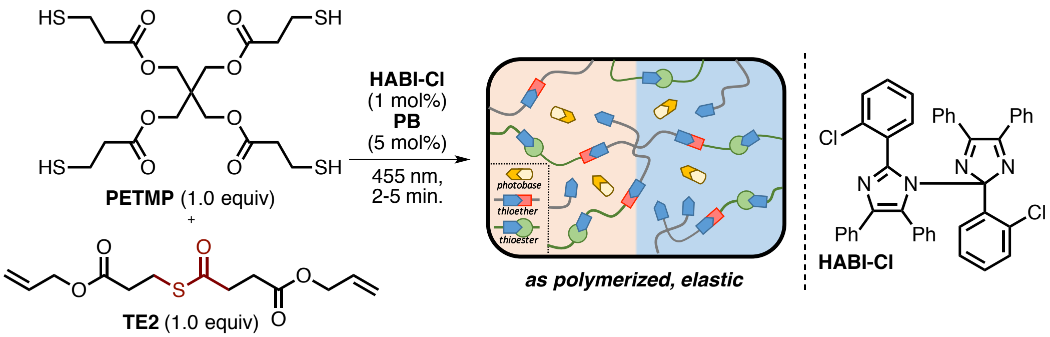

Supplementary Figure 3: A representative procedure for the preparation of a thioester network containing a UV-releasable base (solid to fluid phase transition, Figure 2A – D).

To a 10.0 mL speed mixer vial was added 250 mg (0.87 mmol, 1.00 equiv) of **TE2**, 427 mg (0.87 mmol, 1.00 equiv, "100% excess thiol") of pentaerythritol tetra(3-mercaptopropionate) (PETMP), and 14.0 mg (0.04 mmol, 0.05 equiv, 5.00 mol%) of NPPOC-TMG (**PB**). This suspension was then manually mixed with a pipette tip for ~10 minutes with mild heating and sonication to make a completely homogenous mixture. Following this, approximately 5.73 mg ($8.70\text{x}10^{-3}$ mmol, 0.01 equiv, 1.00 mol%) of **HABI-Cl** was added and the resin was placed onto a hot plate set at 60°C for ~10 minutes with sporadic manually mixing using a pipette tip until most of the **HABI-Cl** had dissolved. At this time the cloudy yellowish resin was filtered through a cotton plugged Pasteur pipette onto a glass slide treated with Rain-X (ITW Global Brands, Houston, TX) using 250 µm thick spacers (Small Parts Inc., Logansport, IN) which was sandwiched between another Rain-X treated glass slide. The material was irradiated (455 nm, 30.0 µW/cm^2, room temperature) for ~2-5 minutes to give the photobase containing network polymer. The conversion was found to be essentially quantitative by IR, revealing complete consumption of the "ene" species while an evident concentration of thiol remained (**S13**).

***A representative experimental setup/procedure for the temporally controlled stress relaxation of a thioester containing network polymer with a UV-releasable base* (solid to fluid phase transition, Figure 2D)**:

A small strip of fully cured material formed from a photo-initiated thiol-ene polymerization (preparation detailed above) was placed onto the DMA (TA Instruments RSA-G2), and a light guide equipped with a collimating lens attached to a mercury lamp (365 nm filter) was placed in close proximity (~5.00 cm) to the front side of the sample. A stress relaxation experiment (10% strain) was started and irradiation (365 nm, 75.0 mW/cm^2, room temperature) of the sample took place 5 minutes, 10 minutes, or 15 minutes after the experiment had begun. In each case the sample was continuously irradiated, once started, for the duration of the experiment (30 minutes total). To obtain the control, simply the sample was not irradiated, and data was collected for 30 minutes.

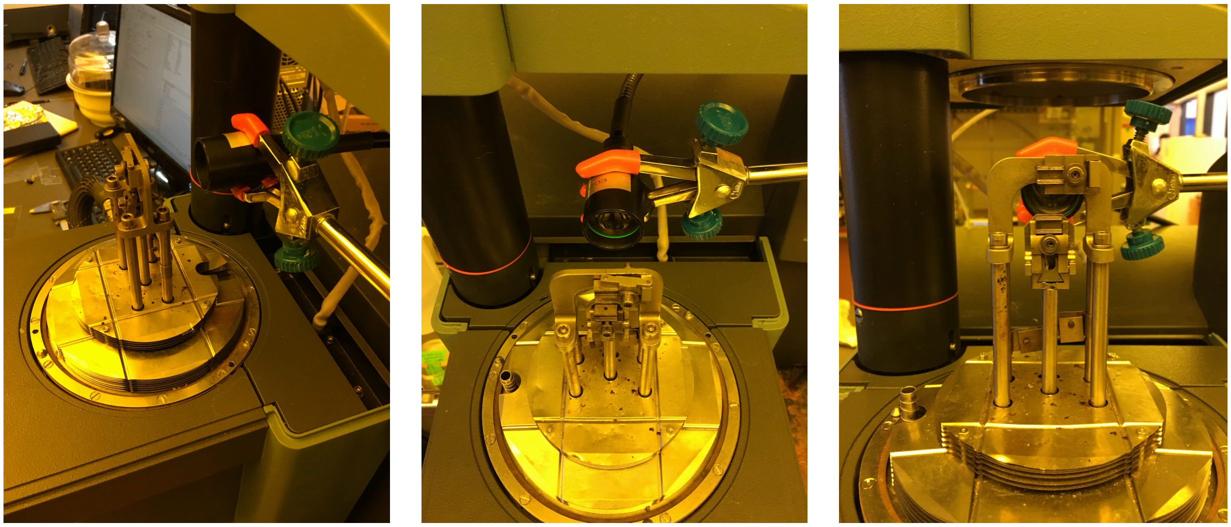

side *top* *front*

Supplementary Figure 4: DMA setup for solid to fluid phase transition.

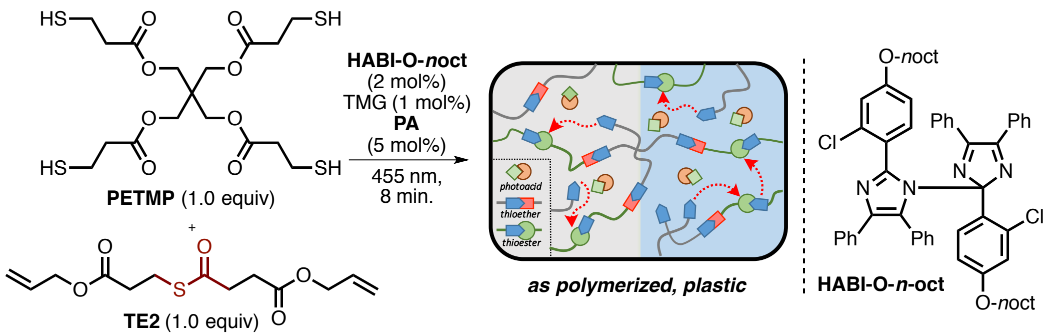

Supplementary Figure 5: A representative procedure for the preparation of a thioester network containing a UV-releasable acid (fluid to solid phase transition, Figure 3A – D).

To a 10.0 mL speed mixer vial was added 500 mg (1.74 mmol, 1.00 equiv) of **TE2**, 849 mg (1.74 mmol, 1.00 equiv, "100% excess thiol") of pentaerythritol tetra(3-mercaptopropionate) (PETMP), 2.20 μLs (2.02 mg, 1.74×10^{-2} mmol, 0.01 equiv, 1.00 mol%) of TMG, and 26.0 mg (8.70×10^{-2} mmol, 0.05 equiv, 5.00 mol%) of NPPOC-phenylacetic acid (NPPOC-PAA, **PA**). This suspension was then manually mixed with a pipette tip for ~5 minutes with mild heating and vortexing to make a completely homogenous mixture. Following this, approximately 63.6 mg (6.96×10^{-2} mmol, 0.04 equiv, 4.00 mol%) of **HABI-O-*n*-oct** was added and the resin was further manually mixed with a pipette tip for an additional ~5 minutes to form a homogeneous mixture (due to the increased solubility of **HABI-O-*n*-oct** in the resin, when compared to **HABI-Cl**, only mild, intermediate heating and vortexing was required). At this time the yellow resin was poured between two glass slides treated with Rain-X (ITW Global Brands, Houston, TX) using 250 μm thick spacers (Small Parts Inc., Logansport, IN). The material was irradiated (455 nm, 50.0 μW/cm^2, room temperature) for 8 minutes (4 minutes each side, timed) to give the photoacid containing network polymer. The conversion was found to be essentially quantitative by IR, revealing complete consumption of the "ene" species while an evident concentration of thiol remained (**S19**).

***A representative experimental setup/procedure for the temporally controlled stress relaxation of a thioester containing network polymer with a UV-releasable acid* (fluid to solid phase transition, Figure 3D)**:

A small strip of fully cured material formed from a photo-initiated thiol-ene polymerization (preparation detailed above) was placed onto the DMA (TA Instruments RSA-G2) and a light guide equipped with a collimating lens attached to a mercury lamp (320-500 nm filter) was placed in close proximity (~5.00 cm) to the sample. A stress relaxation experiment was started and irradiation (320-500 nm, 75.0 mW/cm^2, room temperature) of the samples took place 5, 20, and 60 seconds after the stress experiment had started. In each case the sample was continuously irradiated for 120 seconds and the light was turned off after this period. The stress relaxation experiment was then run the remainder of the period (15 minutes total).

Note: we found that these samples aged significantly, decreasing in the rate of stress relaxation as a function of time once polymerized. Therefore, only a small portion of the material was polymerized at a time (placing the remaining bulk of the un-polymerized material into a -20°C freezer). A static 10-minute aging period following polymerization was employed to allow for homogeneous aging across multiple runs. The sample was loaded onto the DMA during this aging period and the stress relaxation experiment was started promptly at the end of this phase. Any leftover polymerized material was discarded. This procedure was repeated several times to obtain the data shown in **Figure 3D**, placing the bulk of the un-polymerized material back into the -20°C freezer in-between use.

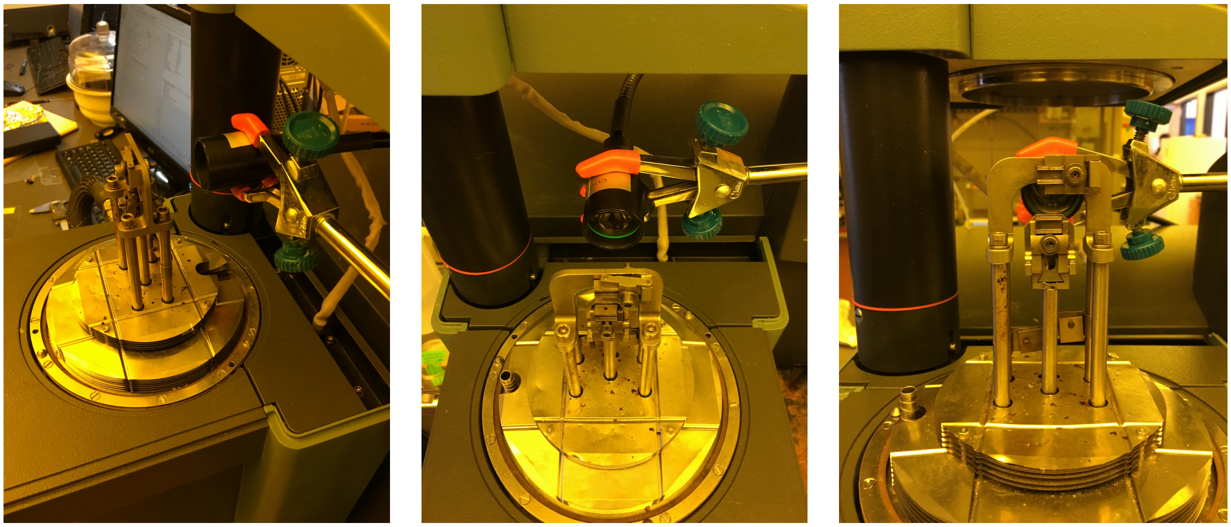

side *top* *front*

Supplementary Figure 6: DMA setup for fluid to solid phase transition.

***A representative experimental setup/procedure for spatial control over nanoindentation* (solid to fluid phase transition, Figure 4A - *top*)**:

The sample was prepared as detailed above in: "*A representative procedure for the preparation of a thioester network containing a UV-releasable base* (solid to fluid phase transition, Figure 2A – D)".

Once prepared, the sample was placed in contact with the positive of a Ralphie^{TM} photomask (dimensions: 32×32 mm, prepared from printing on a polyester transparency film and quadrupling the thickness by stacking and taping 4 cut photomasks together; feel free to contact brady.worrell@gmail.com for additional information, graphic files, or photomask samples) and irradiated for 5 minutes (300-400 nm, 50 mW/cm^2, room temperature). The photomask was removed and the sample was placed in contact with a nanopatterned silicon stamp (LightSmyth, SNS-C12-2525-200-P, period – 833.3 nm, groove depth – 200 nm, line width – 416 nm), loaded into the Nano Imprint Lithography System and compressed from the bottom for 5 minutes (3 MPa). The sample was then removed from the instrument, peeled away from the silicon stamp, and photographed immediately. AFM was performed on the sample (DI 3100, Bruker).

***A representative experimental setup/procedure for spatial control over nanoindentation* (fluid to solid phase transition, Figure 4A - *bottom*)**:

The sample was prepared as detailed above in: "*A representative procedure for the preparation of a thioester network containing a UV-releasable acid* (fluid to solid phase transition, Figure 3A – D)".

Once prepared, the sample was equipped with the positive of a Ralphie^{TM} photomask (dimensions: 32×32 mm, prepared from printing on a polyester transparency film and quadrupling the thickness by stacking and taping 4 cut photomasks together; feel free to contact brady.worrell@gmail.com for additional information, graphic files, or photomask samples) and irradiated for 5 minutes (300-400 nm, 50 mW/cm^2, room temperature). The photomask was removed and the sample was placed in contact with a nanopatterned silicon stamp (LightSmyth, SNS-C12-2525-200-P, period – 833.3 nm, groove depth – 200 nm, line width – 416 nm), loaded into the Nano Imprint Lithography System and compressed from the bottom for 15 minutes (3 MPa). After this period, the sample was irradiated from the top for 5 minutes (300-400 nm, 20 mW/cm^2) while remaining compressed and in contact with the stamp. The sample was then removed from the instrument, peeled away from the silicon stamp, and photographed immediately. AFM was performed on the sample (DI 3100, Bruker).

***A representative experimental setup/procedure for spatially turning on plasticity on a microscopic scale* (solid to fluid phase transition, Figure 4B - *top*)**:

The sample was prepared as detailed above in: "*A representative procedure for the preparation of a thioester network containing a UV-releasable base* (solid to fluid phase transition, Figure 2A – D)".

Once prepared, the sample was cut into a small rectangle (6×15×0.25 mm) and taped to a stretching device using clear Scotch tape with a gap of approximately 8 mm and stretched approximately 2 mm (unit the birefringence became clear though cross-polarizers). The stretching device was set at a 45° angle to the crosspolarizers. The buffalo was irradiated for 2 minutes (400 nm, 125 mW/cm^2, room temperature) through a dynamic light projector (Mightex Polygon 400) integrated into the microscope. Following irradiation, the sample was stretched and allowed to "develop" for 15 minutes and returned to the unstretched state, resulting in the buckled image.

***A representative experimental setup/procedure for spatially turning off plasticity on a microscopic scale* (fluid to solid phase transition, Figure 4B – *bottom*)**:

The sample was prepared as detailed above in: "*A representative procedure for the preparation of a thioester network containing a UV-releasable acid* (fluid to solid phase transition, Figure 3A – D)".

Once prepared, the sample was cut into a small rectangle (6×15×0.25 mm) and taped to a stretching device using clear Scotch tape with a gap of approximately 8 mm and stretched approximately 2 mm (unit the birefringence became clear though cross-polarizers). The stretching device was set at a 45° angle to the cross polarizers. The buffalo was irradiated for 2 minutes (400 nm, 125 mW/cm^2, room temperature) through a dynamic light projector (Mightex Polygon 400) integrated into the microscope. The image was "developed" by allowing it to remain in the stretched state for 15 minutes, then releasing.

Supplementary Discussion:

Thiyl radicals were found to not participate in the exchange of thioesters, which is evidenced by the very high kinetics of the thiol-ene reaction (Supplementary Figure 3). Indeed, the presence of the thioester in stoichiometric quantities has no effect on the rate of this reaction. If the thioester were acting as a RAFT reagent or interacting with the thiyl radical concentration, the rate would slow precipitously. Indeed, it has been shown that the thioester functional group is orthogonal to radical induced polymerizations/processes (see: Neindre, M. L., Magny, B., Nicolaÿ, R. Evaluation of thiocarbonyl and thioester moieties as thiol protecting groups for controlled radical polymerization *Polym. Chem.*, **4**, 5577 (2013)). Moreover, if some concentration of thiolate were formed during irradiation, again, the rate of polymerization would be effected (see: Love, D. M., Kim, K., Goodrich, J. T., Fairbanks, B. D., Worrell, B. T., Stoykovich, M. P., Musgrave, C. B., Bowman, C. N. Amine induced retardation of the radical-mediated thiol-ene reaction via the formation of metastable disulfide radical anions *J. Org. Chem.*, **83**, 2912 (2018)).

Supplementary Figures

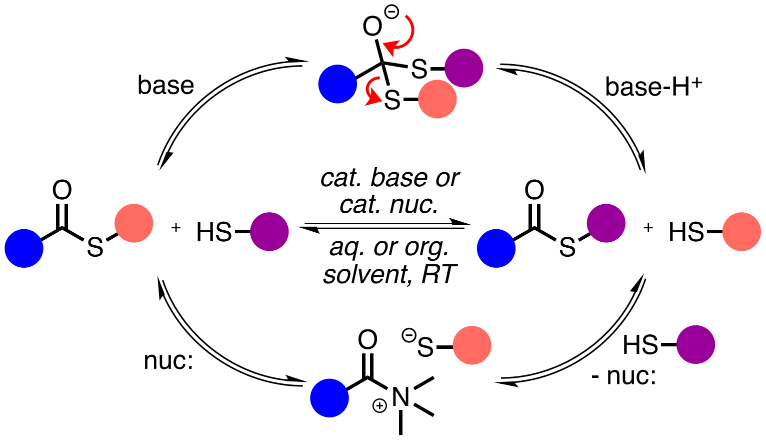

Supplementary Figure 7: Simplified mechanisms for the thiol-thioester exchange catalyzed by bases (*top*) and nucleophiles (*bottom*).

Note: Here (NR_3) as shown on the bottom portion of the graph represents a nucleophile attacking a thioester, not trimethylamine.

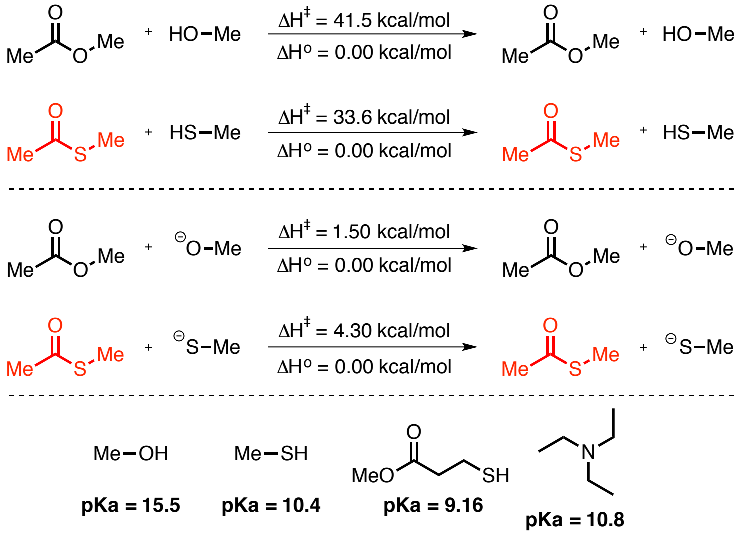

Supplementary Figure 8: Computations reveal that the degenerate exchange of protonated alcohols or thiols with their ester analogs have very high kinetic barriers (*top*), whereas their deprotonated counterparts have very low kinetic barriers (*middle*).

Note: Evidently the thiol-thioester exchange occurs rapidly with mild organic base (such a TEA, pKa = 10.8, conjugate acid in water) due to the significantly lower pKa of thiols when compared to similar alcohols (*bottom*).

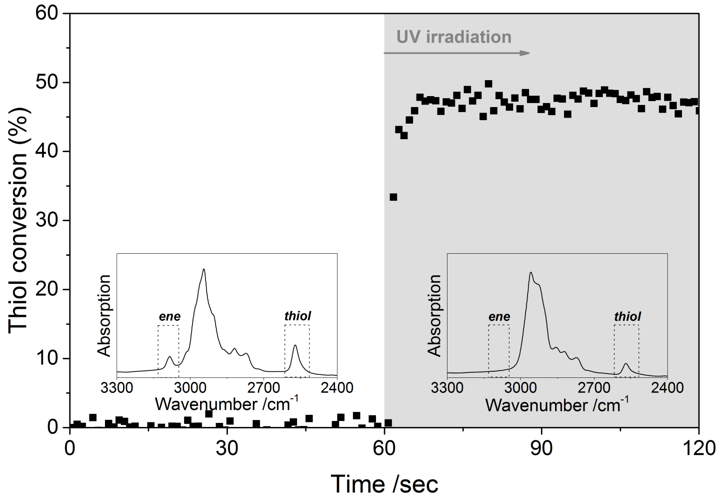

Supplementary Figure 9: The kinetics of the thiol-ene reaction with a thioester containing diene.

General reaction conditions: **TE2** (1.00 equiv), PETMP (1.00 equiv), PMDETA (10.0 mol%), and DMPA (2.00 mol%) monitored by *in situ* IR. Light on at 60.0 seconds and continuously irradiated; 365 nm LED (50.0 mW/cm^2).

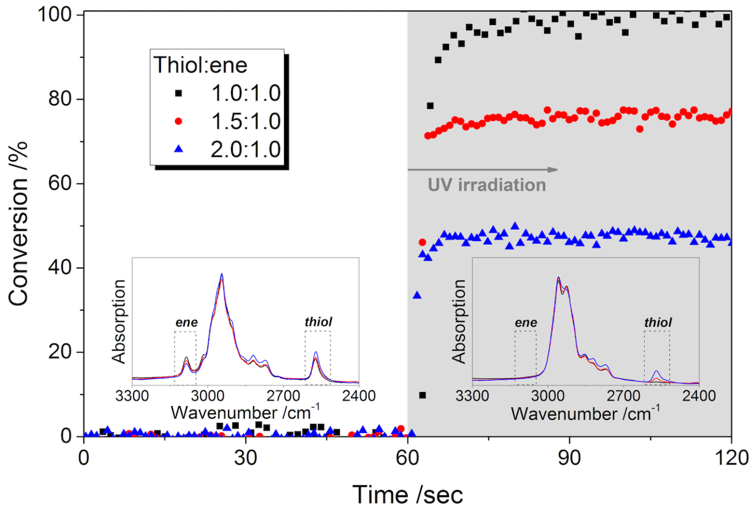

Supplementary Figure 10: Different thiol-ene formulations which show that varying amounts of thiol can remain unreacted in the network based on initial stoichiometry.

General reaction conditions: **TE2** (1.00 equiv [100% xs thiol], 1.50 equiv [50.0% xs thiol], or 2.00 equiv [0.00% xs thiol]), PETMP (1.00 equiv), PMDETA (10.0 mol%), and DMPA (2.00 mol%) monitored by *in situ* IR. Light on at 60.0 seconds and continuously irradiated; 365 nm LED (50.0 mW/cm^2).

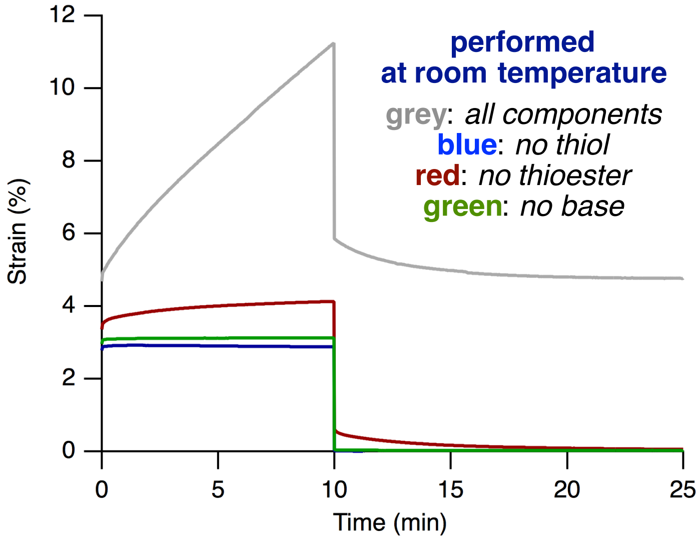

Supplementary Figure 11: Creep compliance showing the requirement for free thiol, thioester, and a basic catalyst in the network for fluidic behavior to occur.

General formulations: *all components* (grey line) – **TE2** (1.00 equiv), PETMP (1.00 equiv), PMDETA (10.0 mol%), and DMPA (2.00 mol%); *w/o thiol* (blue line) – **TE2** (1.00 equiv), PETMP (0.25 equiv), PMDETA (10.0 mol%), and DMPA (2.00 mol%); *w/o thioester* (red line) – **DAEC** (1.00 equiv), PETMP (1.00 equiv), PMDETA (10.0 mol%), and DMPA (2.00 mol%); *w/o base* (green line) – **TE2** (1.00 equiv), PETMP (1.00 equiv), and DMPA (2.00 mol%).

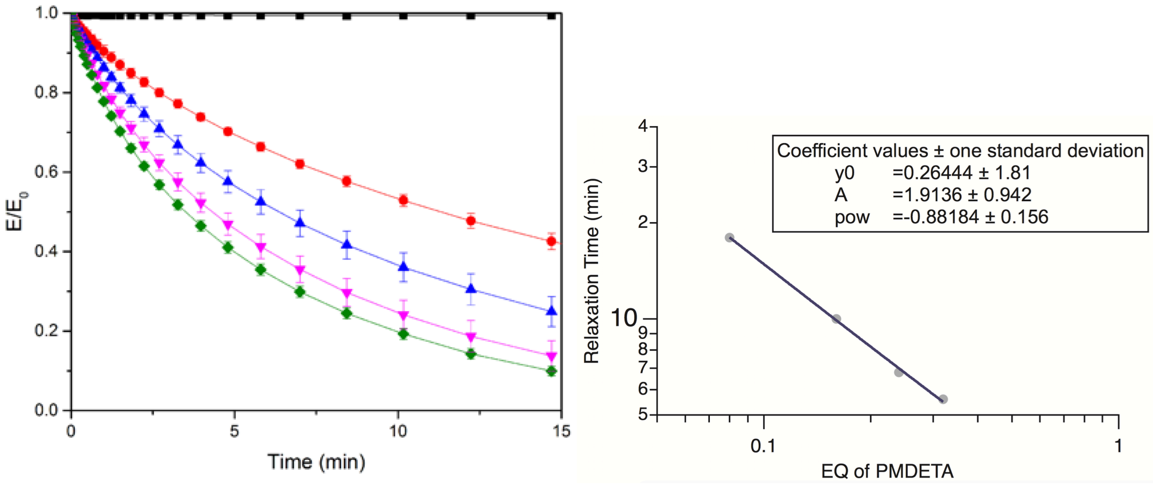

Supplementary Figure 12: Increasing the concentration of PMDETA in the network accordingly increases the rate of stress relaxation (constant applied strain of 10%, *left*). A first order dependence of the base is noted in this system based on the relaxation time for each condition at t = 15 min (*right*).

General formulations: **TE2** (1.00 equiv), PETMP (1.00 equiv), PMEDTA (0.00 mol% [black], 8.00 mol% [red], 16.0 mol% [blue], 24.0 mol% [pink], 32.0 mol% [green]), and DMPA (1.00 mol%). The data shown above is an average of three runs.

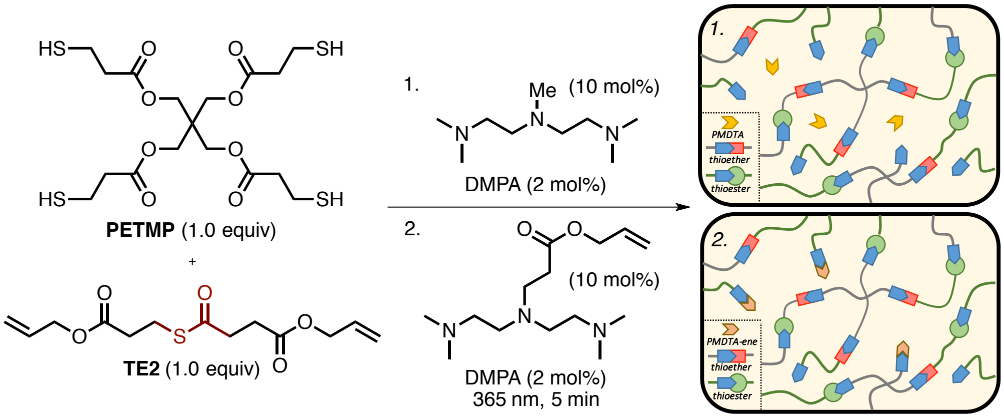

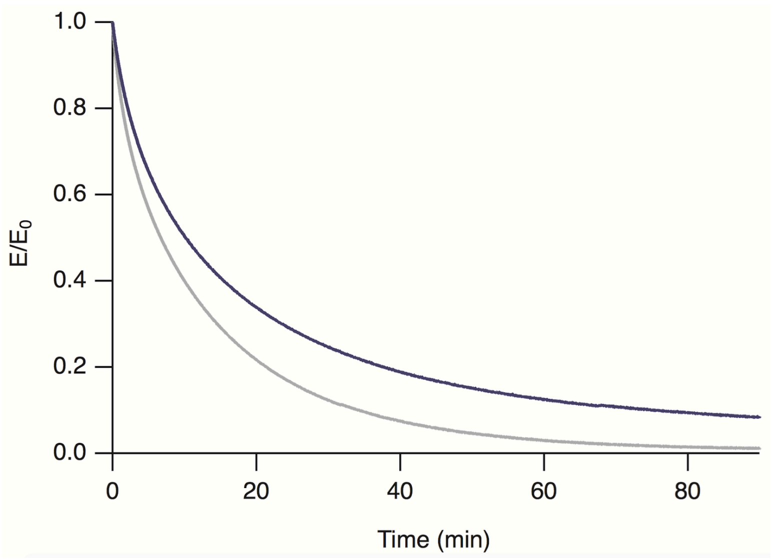

Supplementary Figure 13: Tethering the base to the network evidently does not effect the rate of relaxation when compared to the free base (constant applied strain of 10%).

General formulation: **1**) blue line - **TE2** (1.00 equiv), PETMP (1.00 equiv), PMDETA (10.0 mol%), DMPA (2.00 mol%); **2**) grey line - **TE2** (1.00 equiv), PETMP (1.00 equiv), PMDETA-ene (10.0 mol%), DMPA (2.00 mol%).

Note: although the tethered base gave slightly faster kinetics, the difference is not significant.

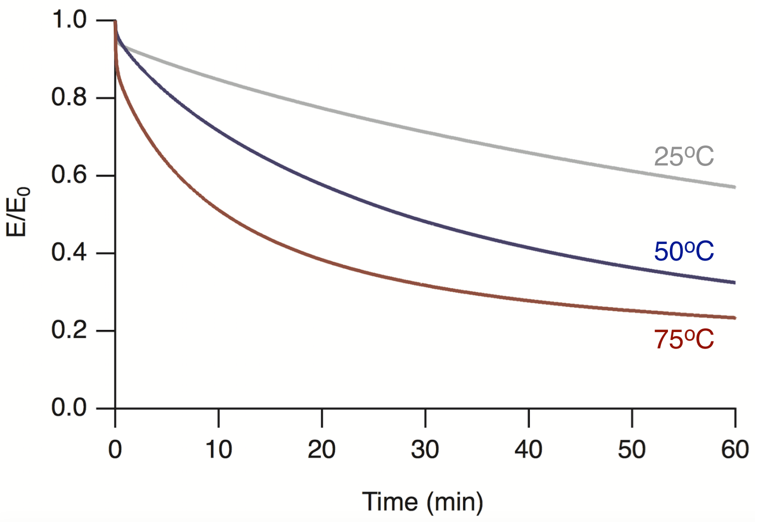

Supplementary Figure 14: Increasing the temperature accordingly increases the rate of stress relaxation with basic catalysts; stress relaxation (constant applied strain of 10%) experiment performed at 25°C, 50°C, and 75°C.

Note: higher crosslink densities were utilized for these experiments. *General formulation*: **TE2** (1.50 equiv), PETMP (1.00 equiv), PMDETA (10.0 mol%), and DMPA (2.00 mol%).

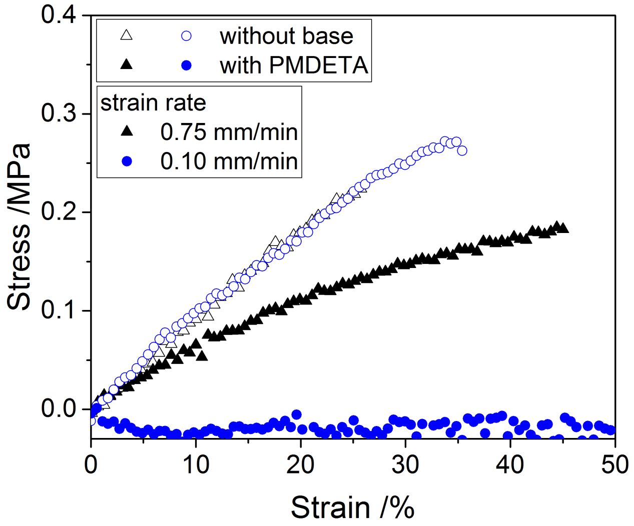

Supplementary Figure 15: Samples with base relax stress when strained (0.75 and 0.10 mm/min); samples without base quickly build up stress (0.75 and 0.10 mm/min). Significantly, when slower strain rates (0.10 mm/min) were applied no stress was built up.

General formulation: **TE2** (1.00 equiv), PETMP (1.00 equiv), PMDETA (0.00 mol% or 10.0 mol%), and DMPA (2.00 mol%).

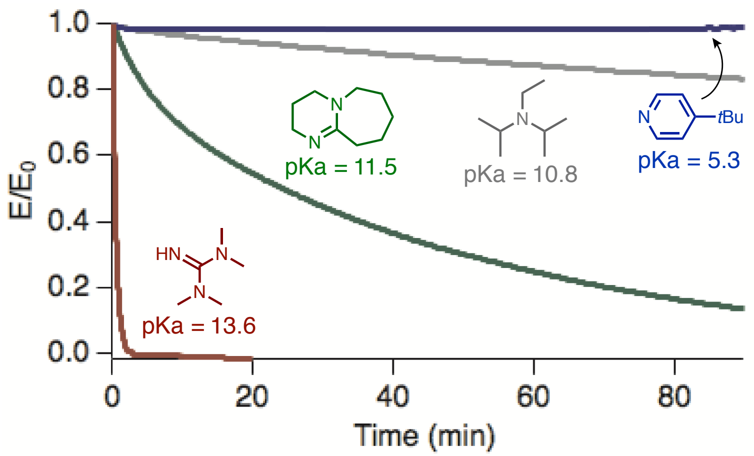

Supplementary Figure 16: Increasing the pKa of the organic base in the network accordingly increases the rate of stress relaxation (constant applied strain of 10%).

General formulations: **TE2** (1.00 equiv), PETMP (1.00 equiv), organic base (3.00 mol%), and DMPA (2.00 mol%).

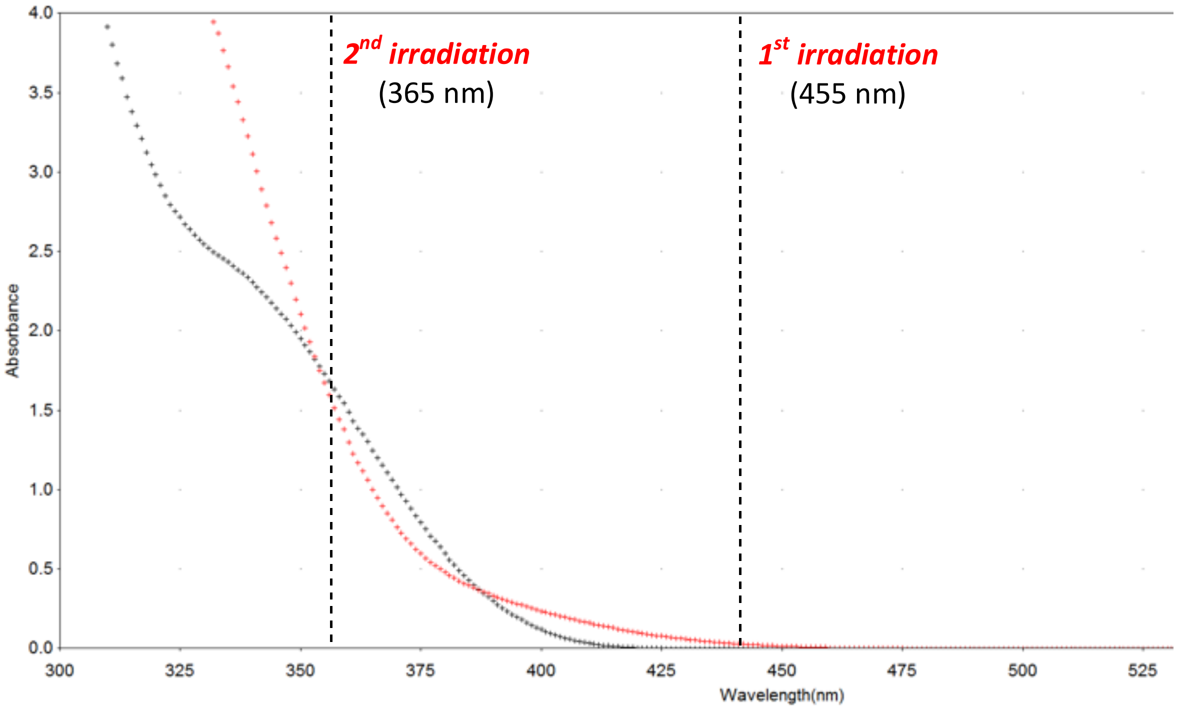

Supplementary Figure 17: Ultraviolet-visible spectra of the photoinitaitor (**HABI-O-*n*-oct**, *red line*) and the photobase (**PB**, NPPOC-TMG, *black line*) in relative concentrations utilized in the ON switch formulation. The photoinitiator has some absorbance at 455 nm where the photobase has essentially none; the photobase strongly absorbs at 365 nm.

Amounts used: *red line* - **HABI-O-*n*-oct** (5.00 mg, 5.47x10^{-3} mmol) in 5.50 mL EtOAc (0.001 M); *black line* - **PB** (5.00 mg, 1.55x10^{-2} mmol) in 3.10 mL EtOAC (0.005 M).

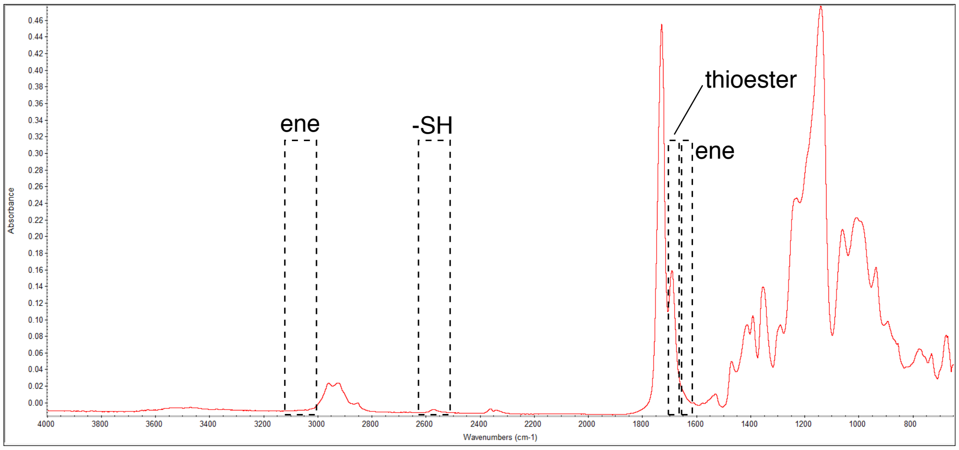

Supplementary Figure 18: ATR showing complete conversion of the thiol-ene reaction with a thioester containing diene in the presence of a UV-labile base following irradiation with 455 nm light, 30.0 mW/cm^2, ~2-3 minutes. No peaks corresponding to ene (~3000 and ~1620 cm^{-1}) were noted, whereas peaks corresponding to thiol (~2550 cm^{-1}) and thioester (1650 cm^{-1}) were still evident.

General formulation: **TE2** (1.00 equiv), PETMP (1.00 equiv), **PB** (5.00 mol%), and **HABI-Cl** (1.00 mol%).

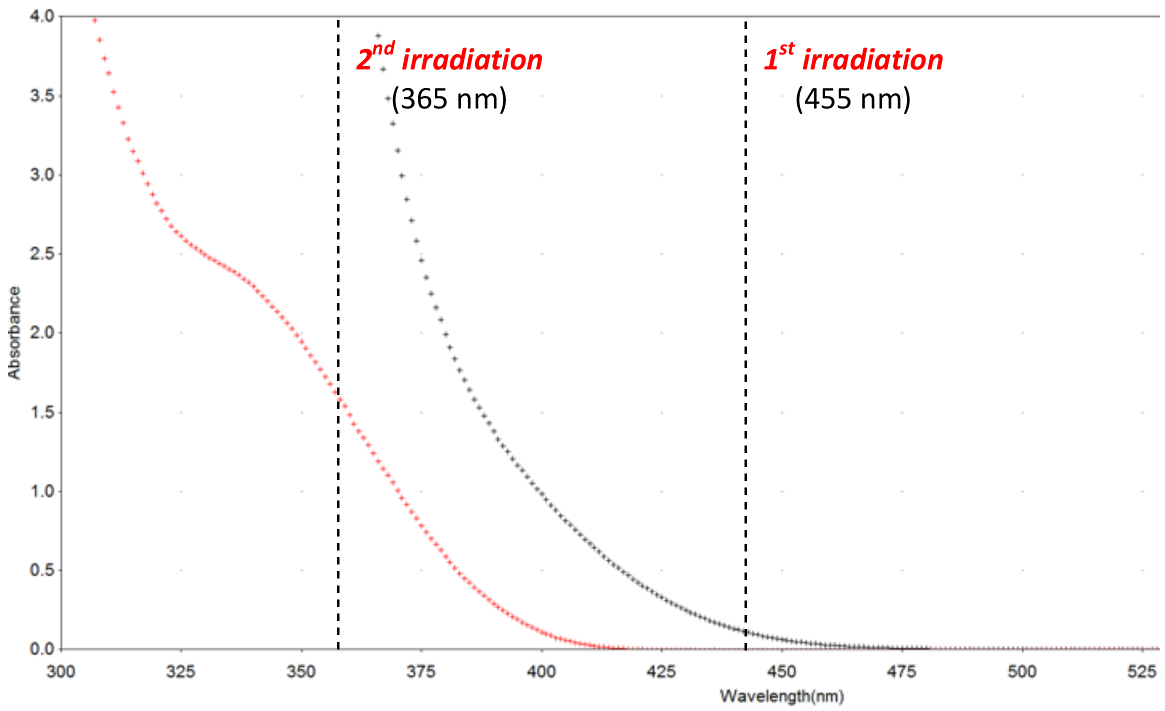

Supplementary Figure 19: Ultraviolet-visible spectra of the photoinitaitor (**HABI-O-*n*-oct**, *black line*) and the photoacid (**PA**, NPPOC-phenylacetic ester, *red line*) in the relative concentrations utilized in the fluid to solid formulation. The photoinitiator has good absorbance at 455 nm where the photoacid has essentially none; the photoacid strongly absorbs at 365 nm.

Amounts used: *black line* - **HABI-O-*n*-oct** (5.00 mg, $5.47x10^{-3}$ mmol) in 1.37 mL EtOAc (0.004 M); *red line* - **PA** (5.00 mg, $1.67x10^{-2}$ mmol) in 3.34 mL EtOAC (0.005 M).

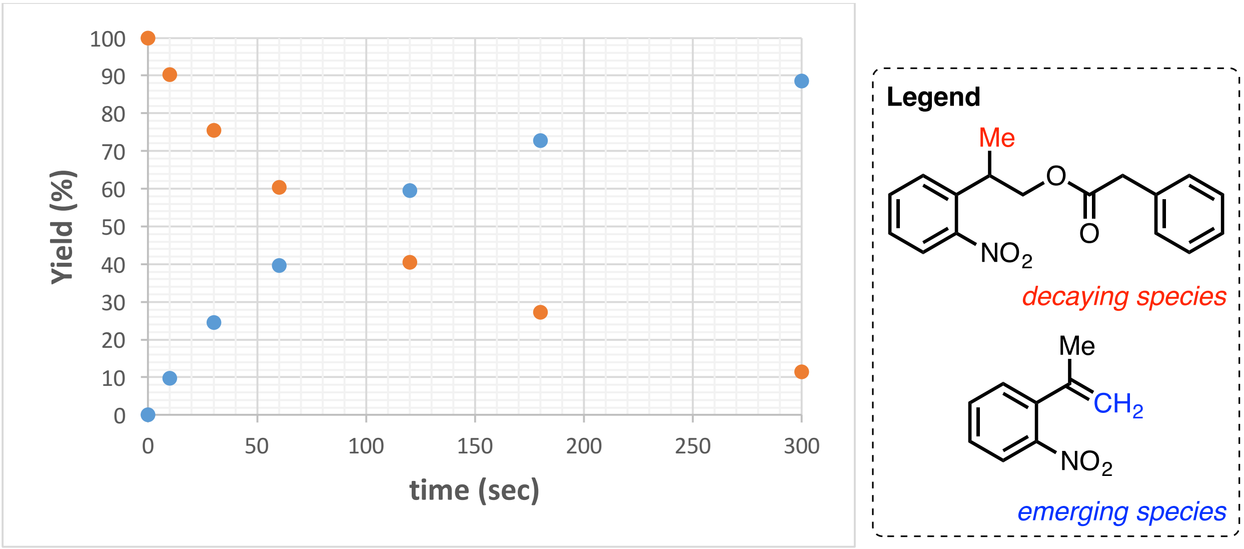

Supplementary Figure 20: Time-course over 5 minutes (300 sec) for the photo-triggered released of phenylacetic acid from **PA** by ^{1}H-NMR.

General reaction conditions: **PA** (1.00 equiv), 1,3,5-trimethoxybenzene (0.50 equiv, *internal standard*), MeCN-d_3 (0.10 M), ~75.0 mW/cm^2, 365 nm, RT.

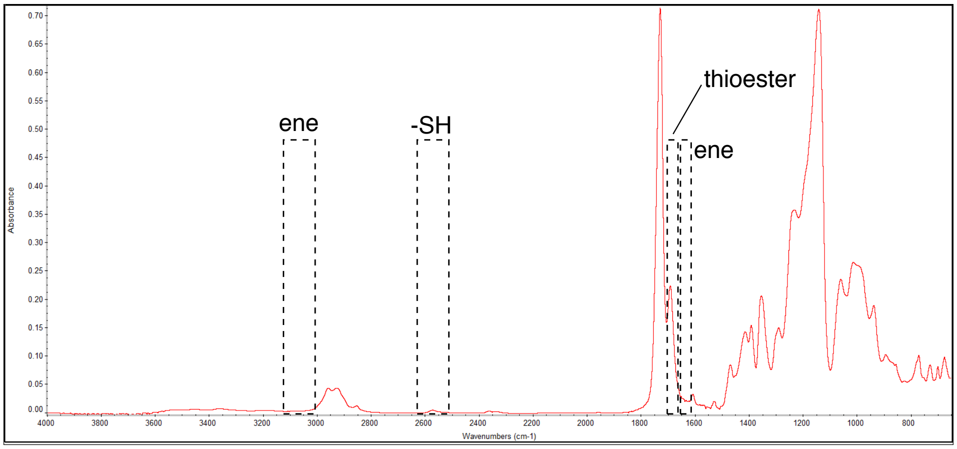

Supplementary Figure 21: ATR showing complete conversion of the thiol-ene reaction with a thioester containing diene in the presence of a UV-labile acid following irradiation with 455 nm light, 30.0 mW/cm^2, 8 minutes (4 minutes per side). No peaks corresponding to ene (~3000 and ~1620 cm^{-1}) were noted, whereas peaks corresponding to thiol (~2550 cm^{-1}) and thioester (1650 cm^{-1}) were still evident.

General formulation: **TE2** (1.00 equiv), PETMP (1.00 equiv), TMG (1.00 mol%), **PA** (5.00 mol%), and **HABI-O-*n*-oct** (4.00 mol%).

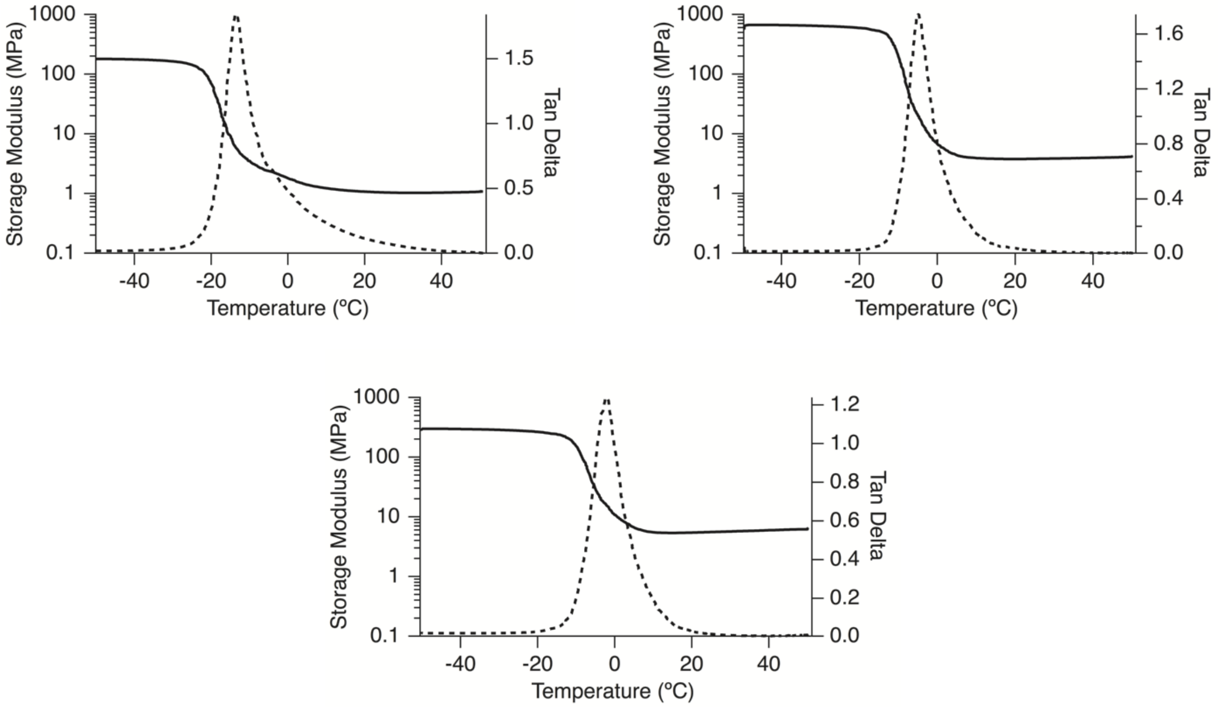

Supplementary Figure 22: Glass transition (T_g) of various polymers utilized in our studies based on different ratios of **TE2** and PETMP (1:1 [*top left*, T_g = -14°C], 1.5:1 [*top right*, T_g = -5°C], and 2:1 [*bottom middle*, T_g = -2°C], molar equivalency) formed by photopolymerization.

General formulation: **TE2** (1.00 [*top left*], 1.50 [*top right*], 2.00 [*bottom middle*]), PETMP (1.00 equiv), DMPA (2.00 mol%).

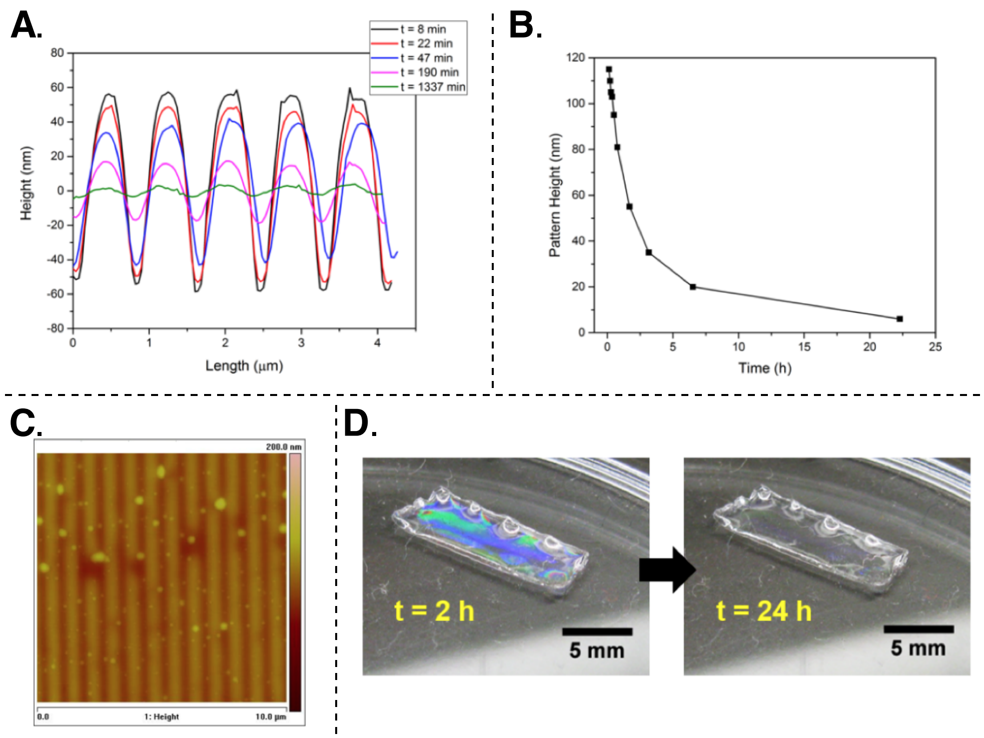

Supplementary Figure 23: Nanoimprint lithography on a thioester-containing elastomer.

The master silicon pattern had a depth of 220 nm and period of 880 nm. Imprinting was performed at 20 bar pressure for 10 minutes close to ambient temperature. **A**) Surface profile of the imprinted surface over time, continuously monitored by contact AFM and analyzed at various time points; **B**) imprinted pattern height changes with elapsed time after imprinting; **C**) 10 x 10 µm area topography of imprinted surface. Surface defects are due to defects in the silicon master; **D**) photographs demonstrating changes in the optical reflective properties of the film over time after imprinting. After 24 hours, the surface pattern had faded to the point where colored reflection was no longer visible at the same viewing angle.

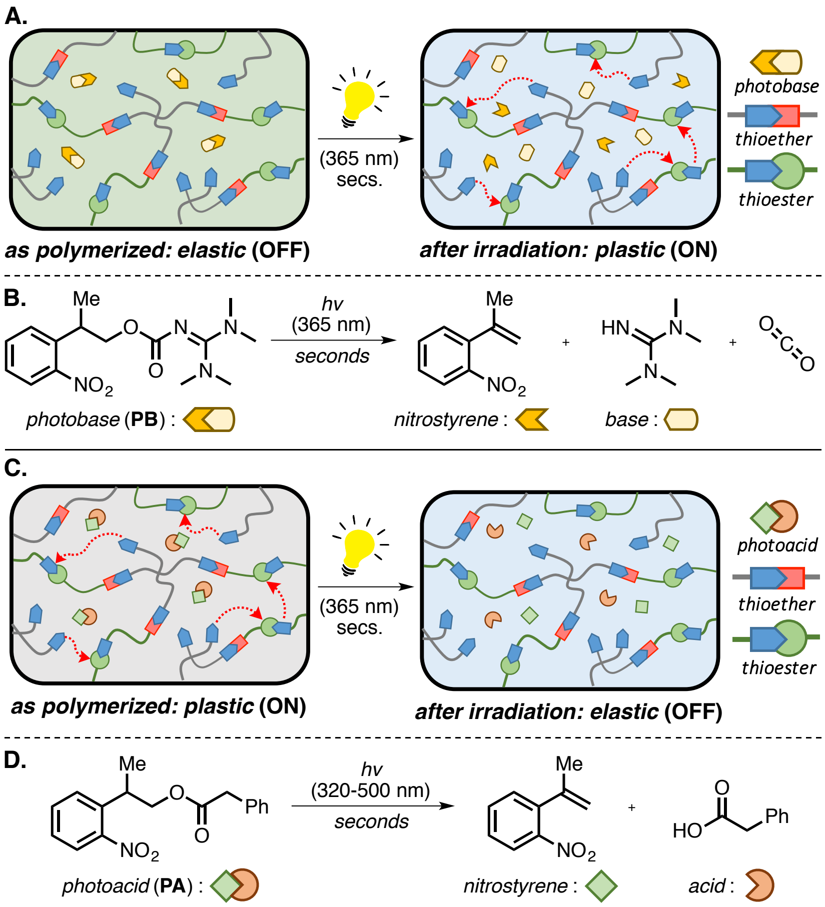

Supplementary Figure 24: Concept of photoswitchable plasticity using photo-releasable bases and acids.

A) A cartoon depicting the OFF to ON switch in plasticity of a photobase containing thioester network polymer; **B**) the photobase when irradiated with UV light exposes the potent base, TMG; **C**) a cartoon depicting the ON to OFF switch in plasticity of a photoacid containing thioester network polymer; **D**) the photoacid when irradiated with UV light exposes the non-volatile acid, phenylacetic acid.

Supplementary Figure 25: ^{1}H and ^{13}C spectra of **TE1**.

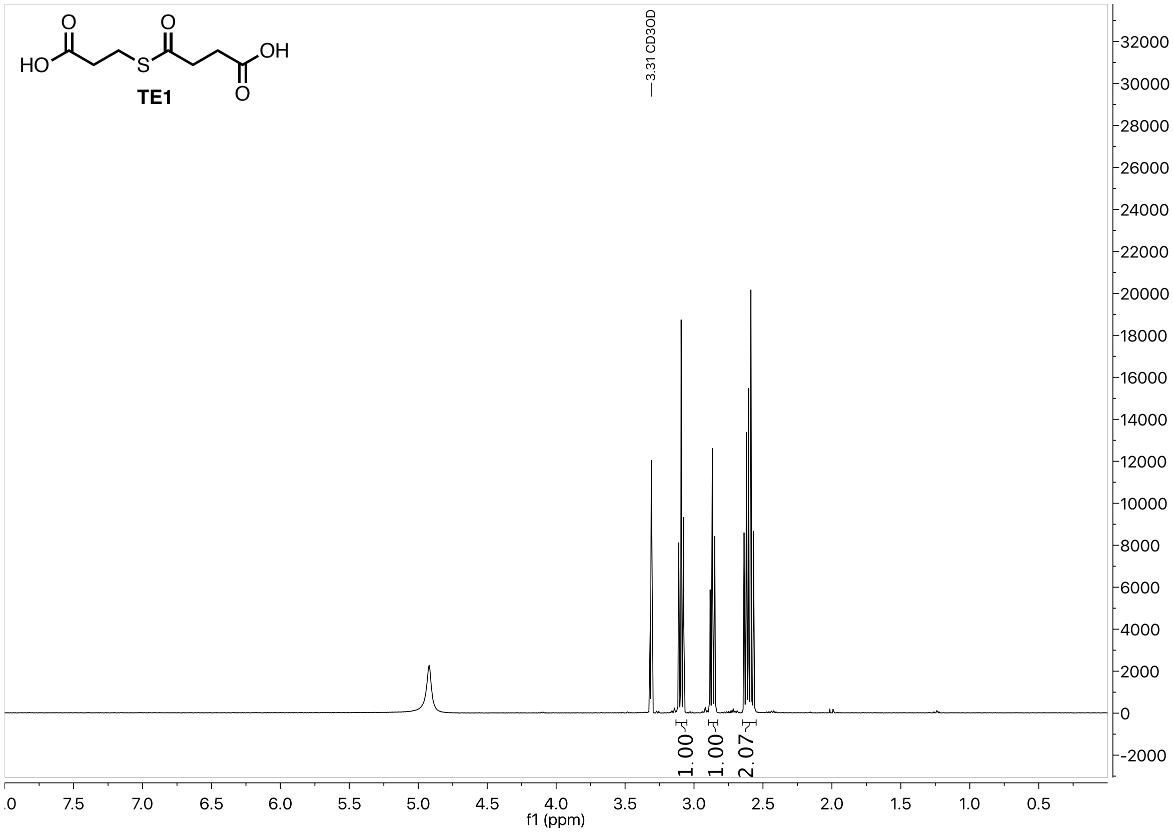

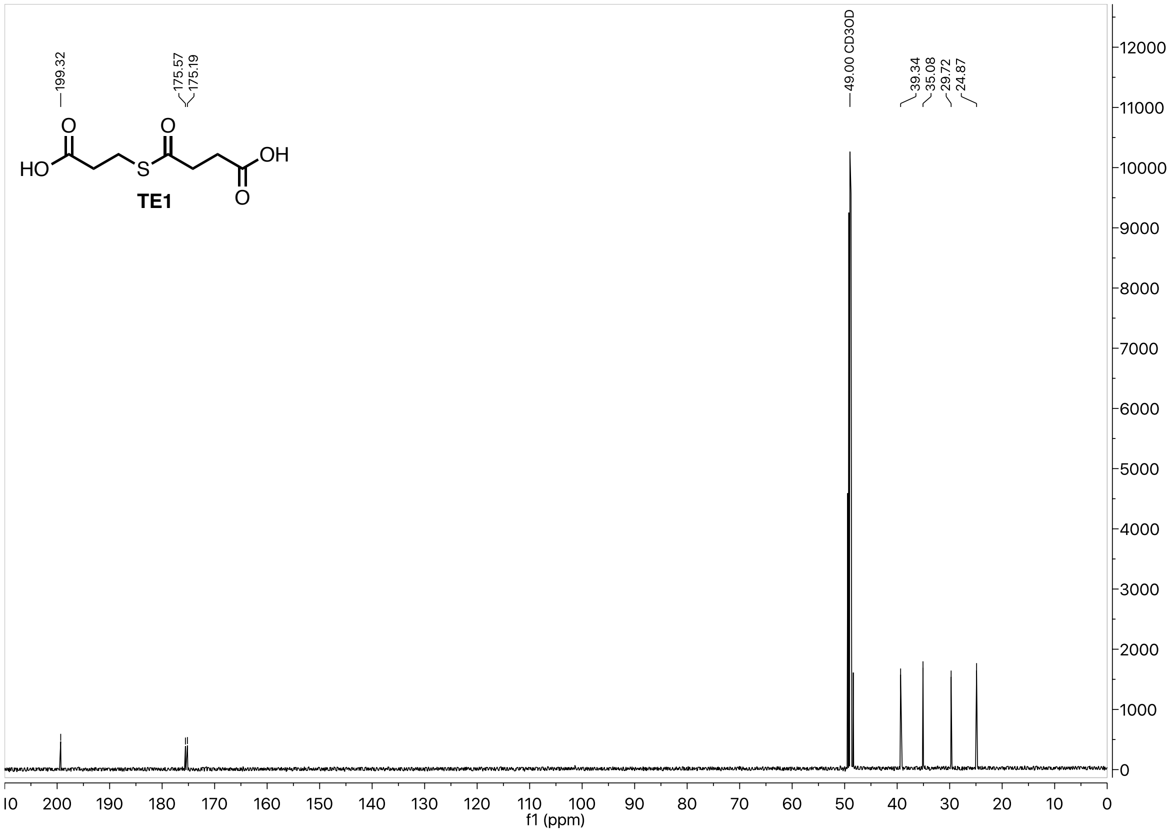

Supplementary Figure 26: ^{1}H and ^{13}C spectra of **TE2**.

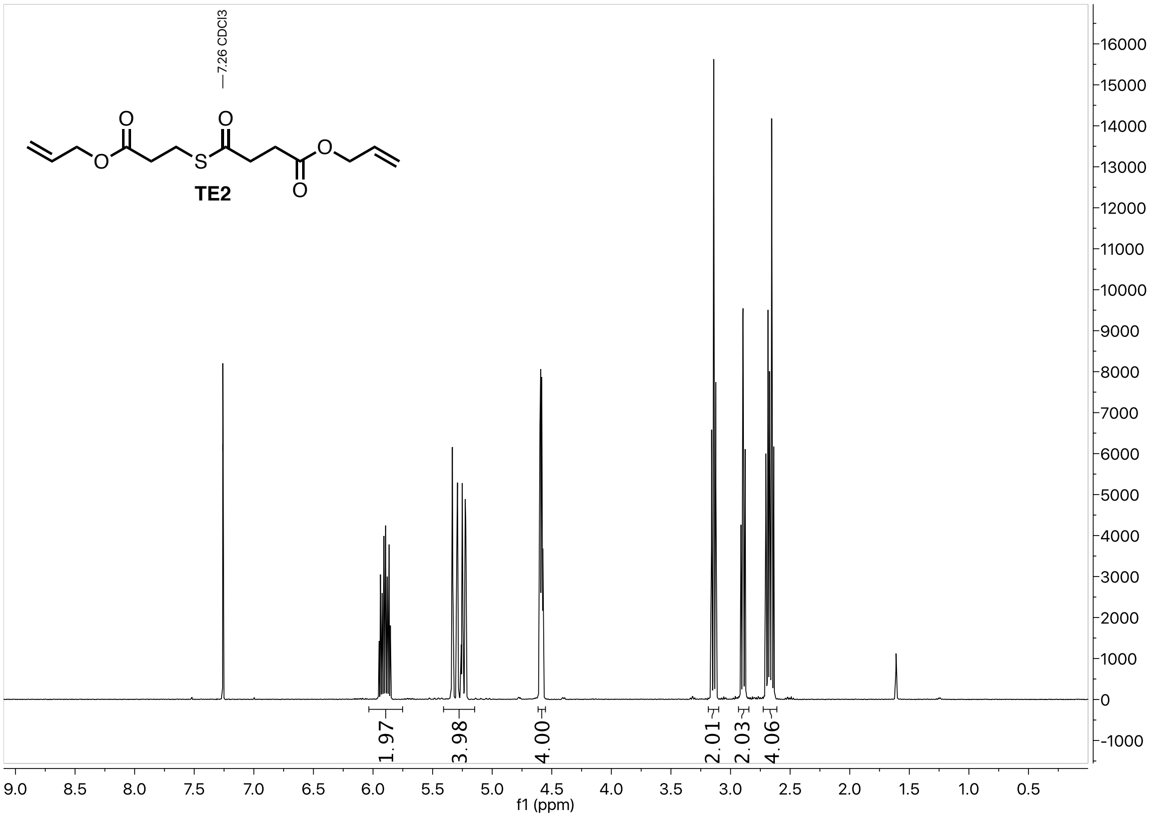

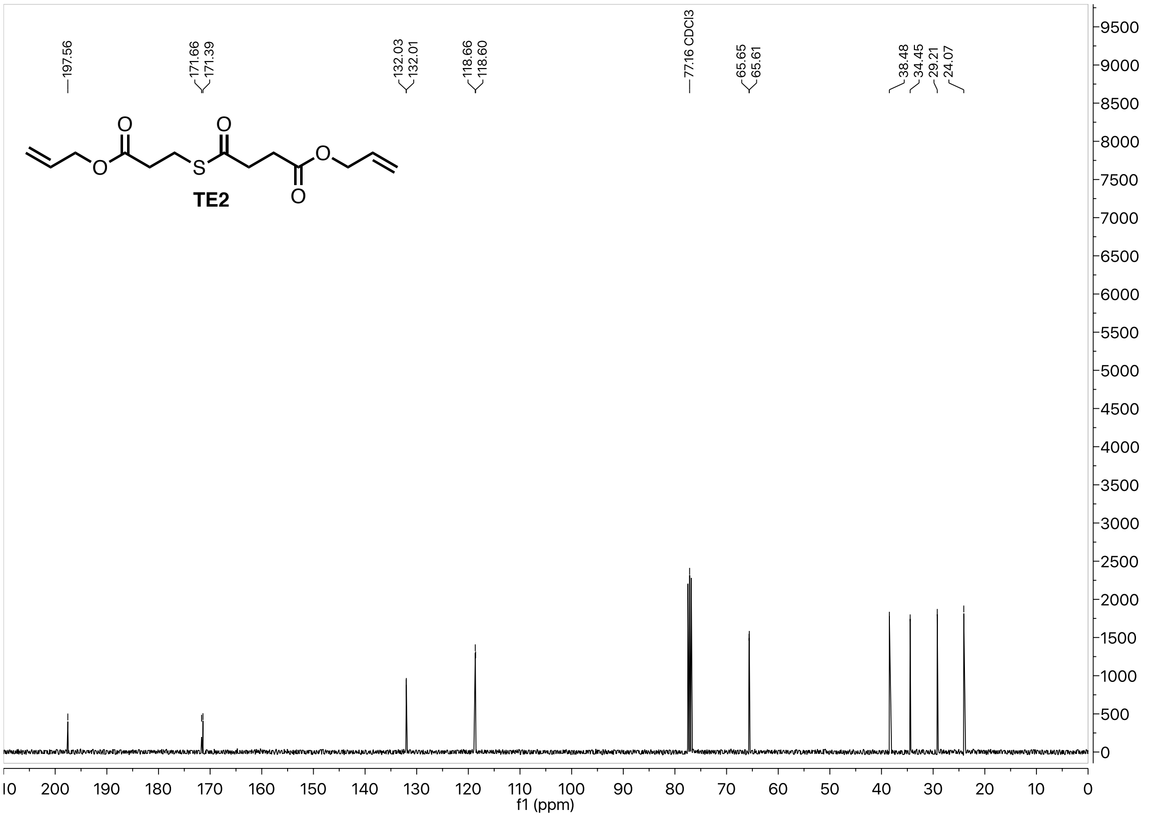

Supplementary Figure 27: ^{1}H and ^{13}C spectra of **DAEC**.

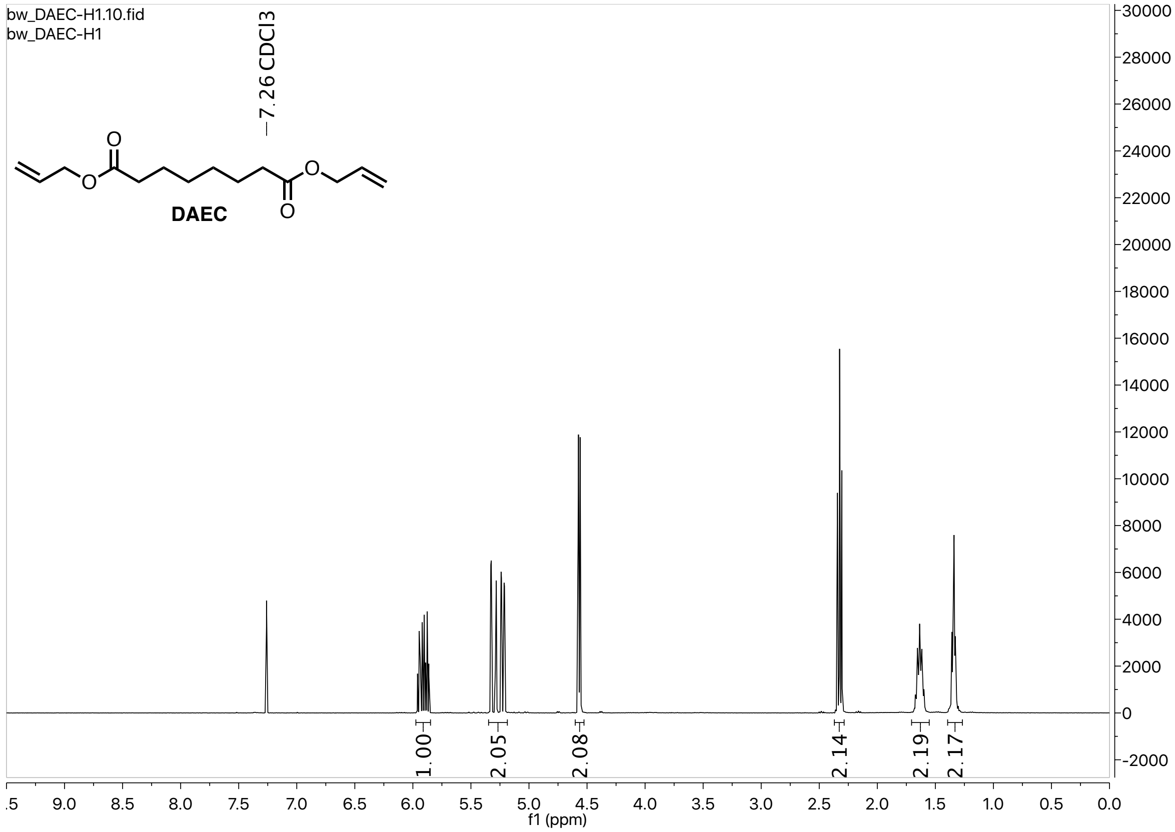

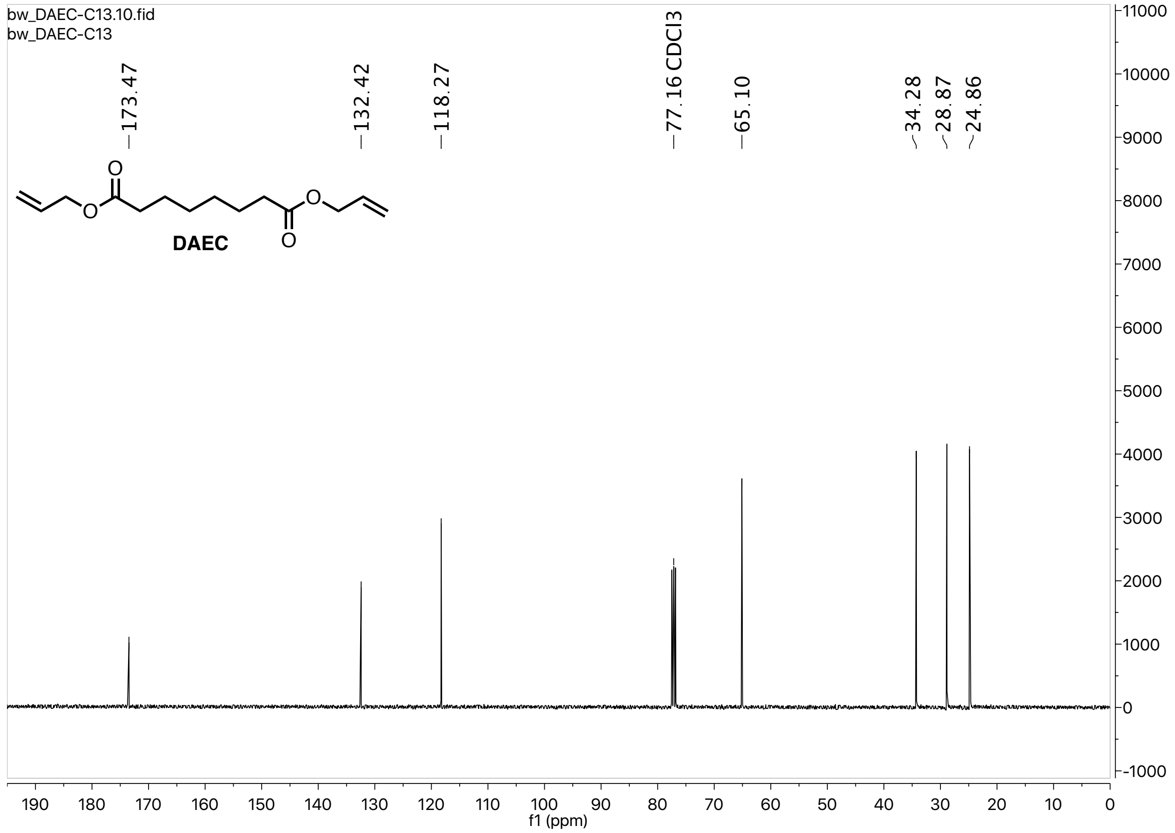

Supplementary Figure 28: ^{1}H and ^{13}C spectra of **PB**.

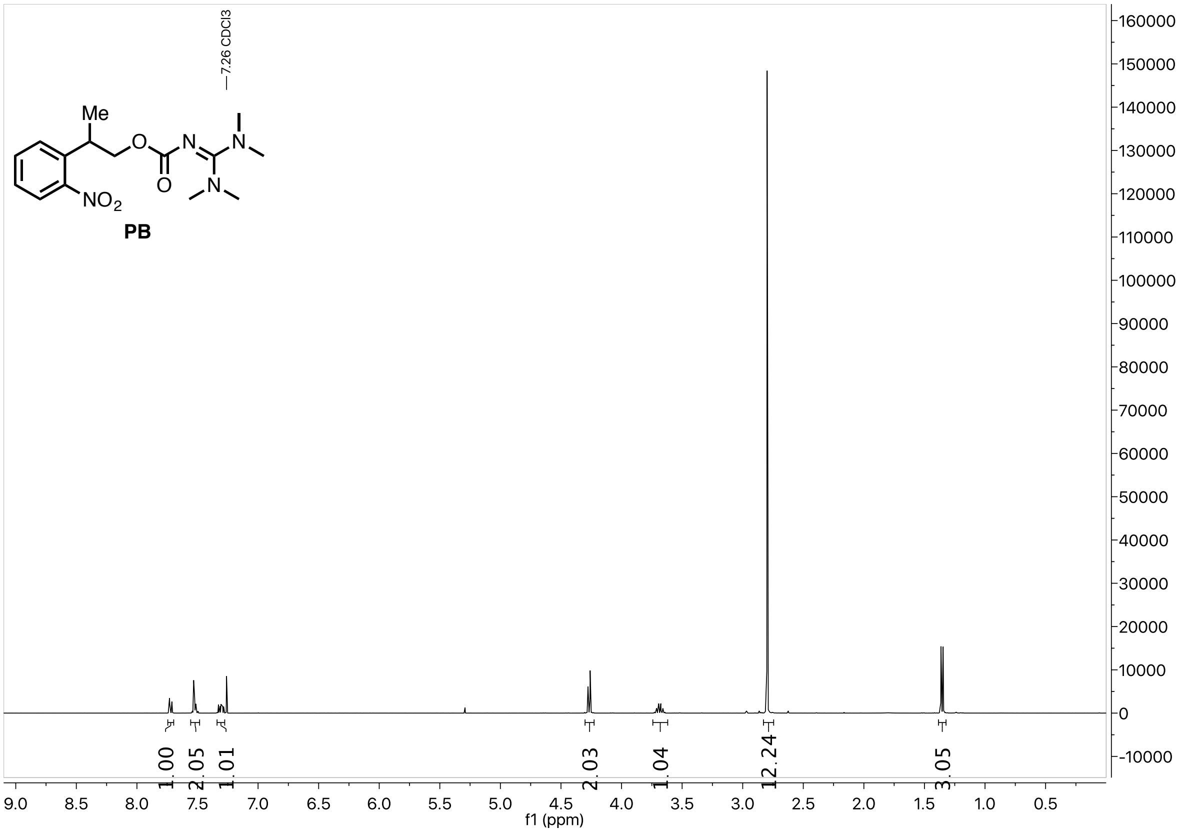

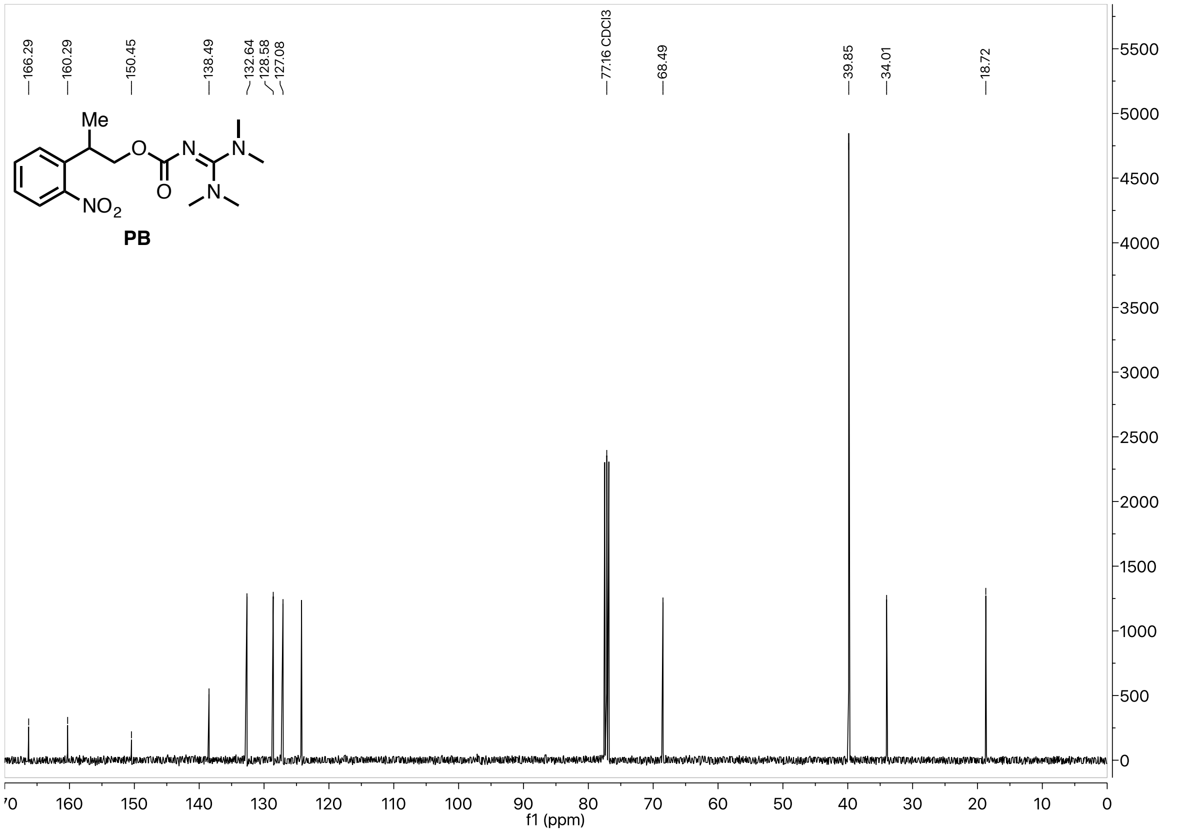

Supplementary Figure 29: ^{1}H and ^{13}C spectra of **HABI-1**.

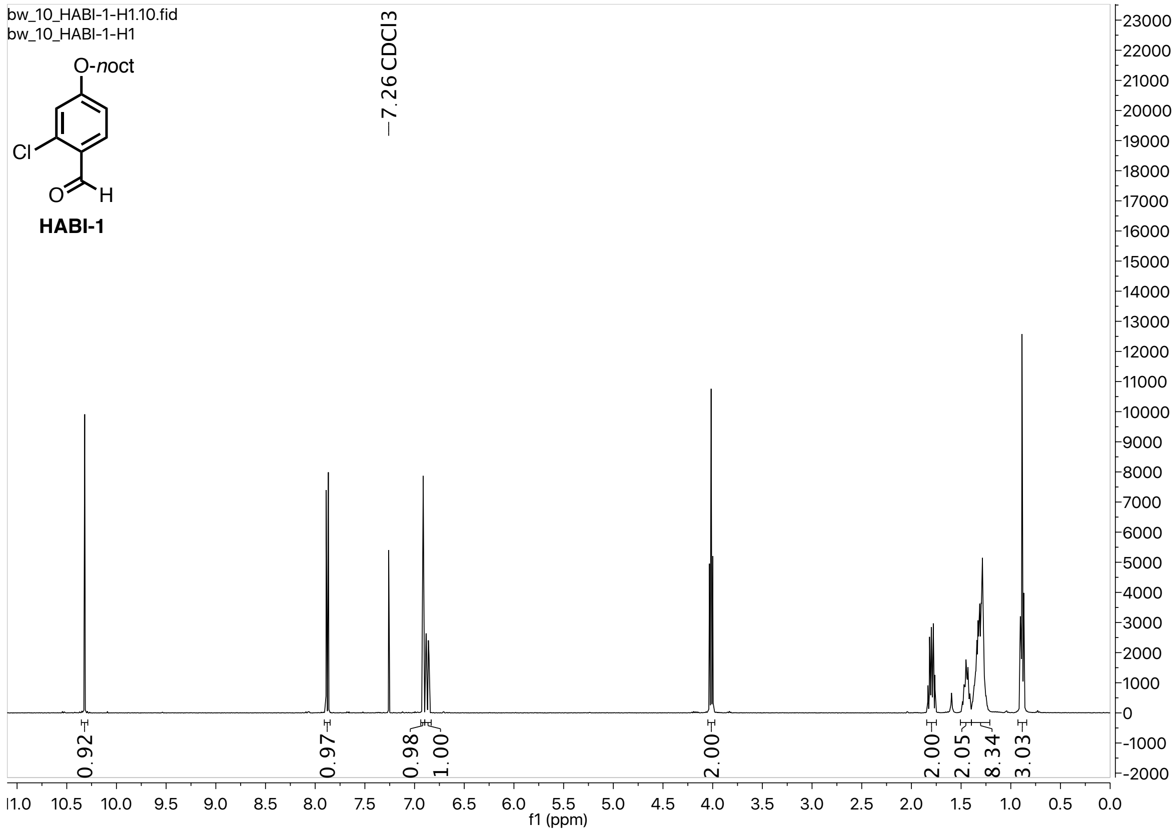

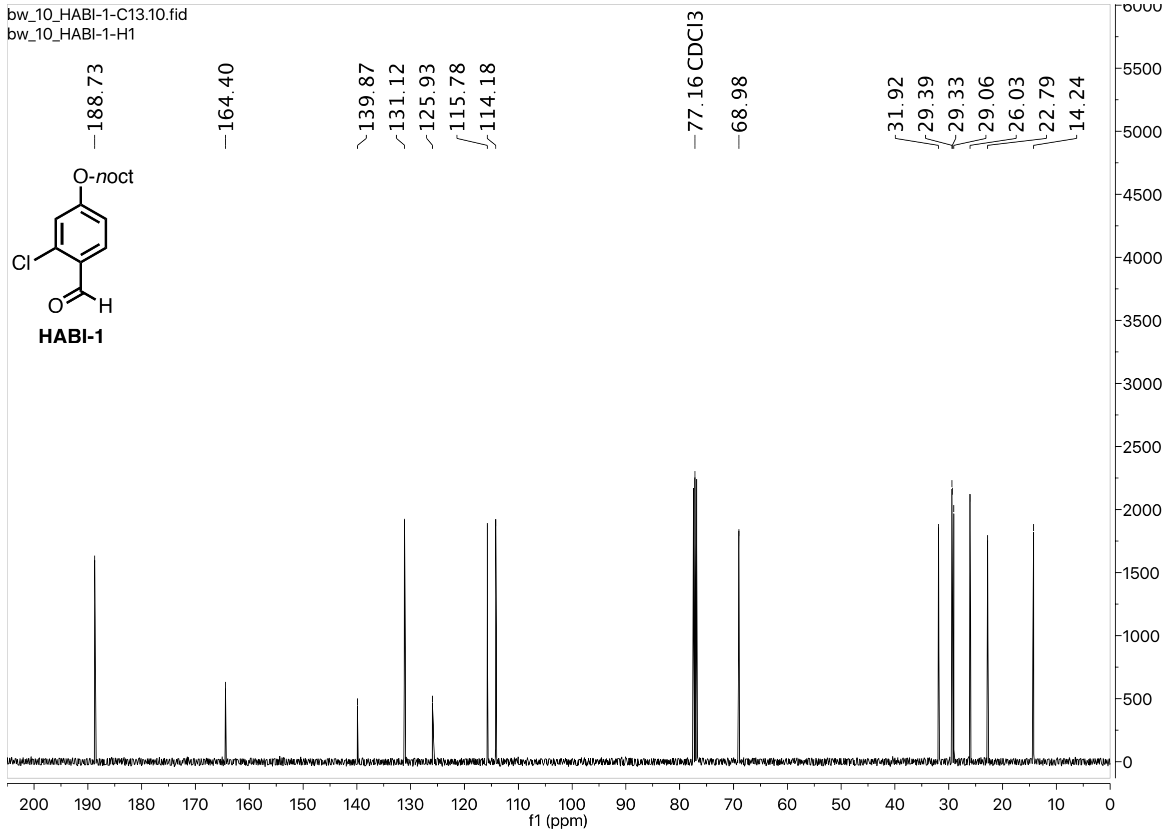

Supplementary Figure 30: ^{1}H and ^{13}C spectra of **HABI-2**.

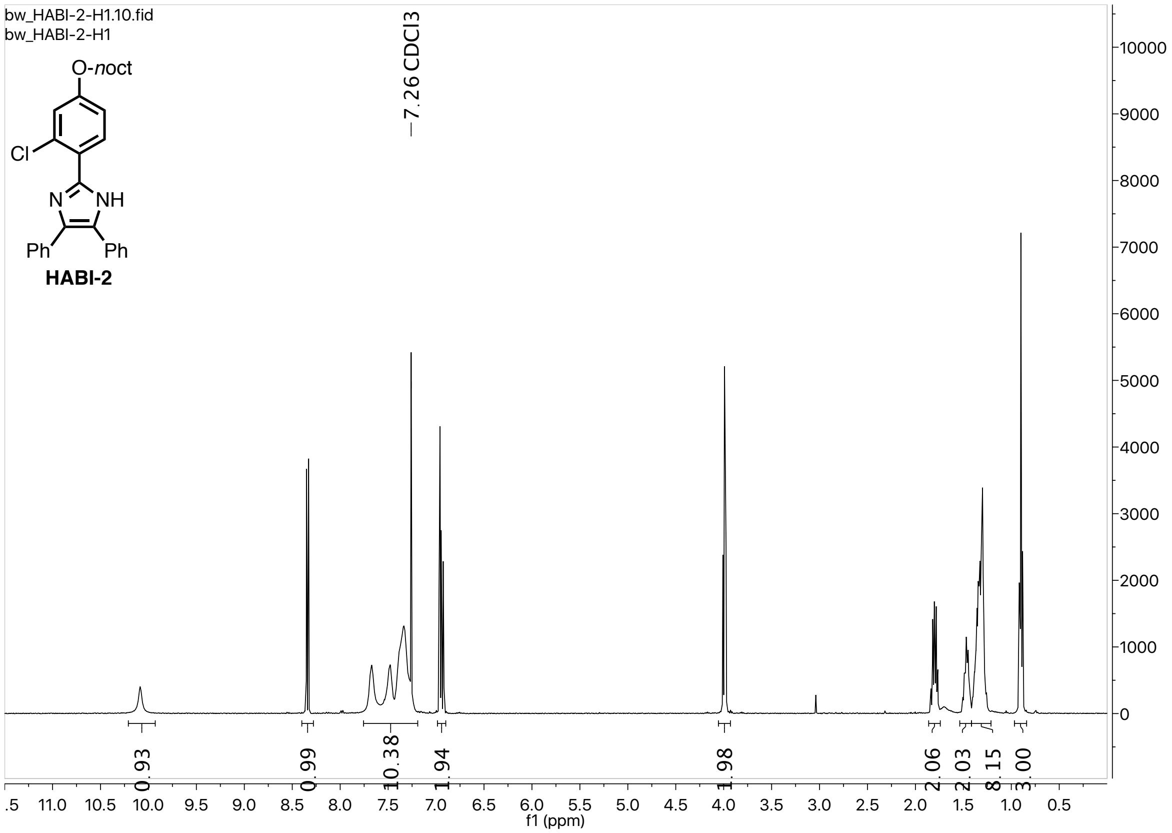

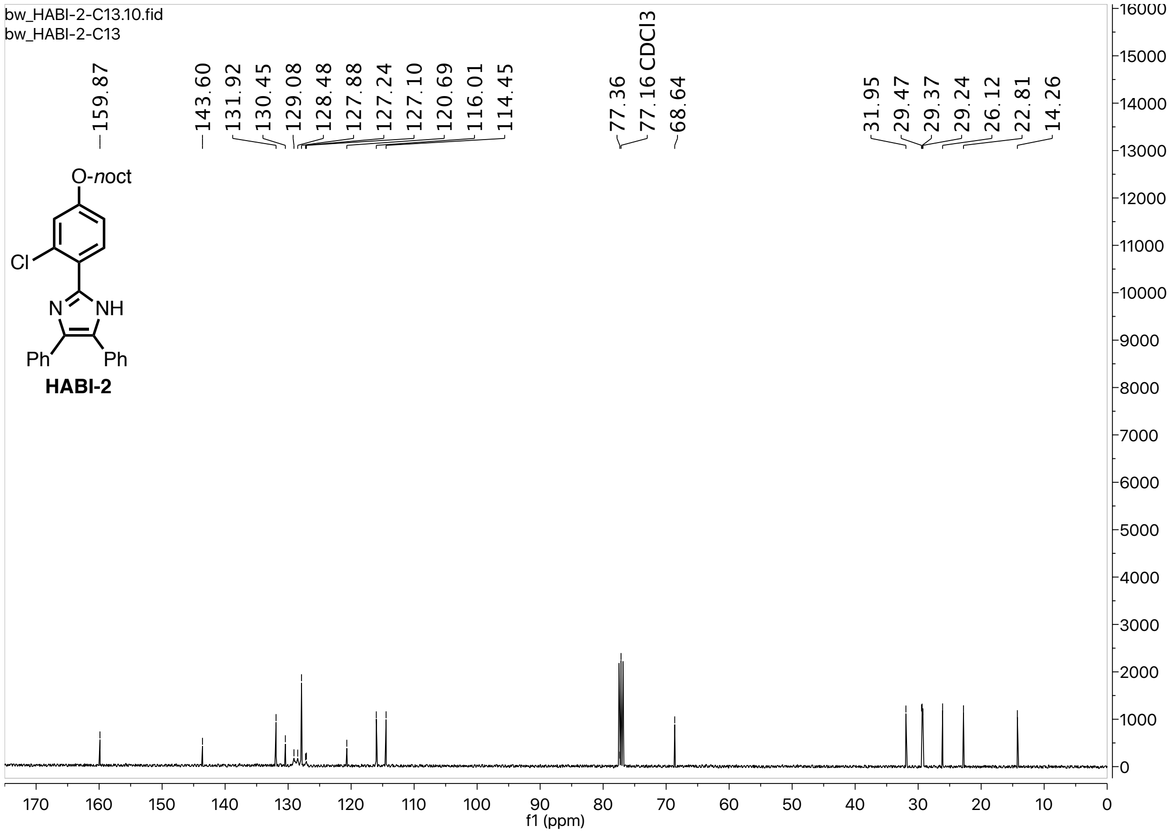

Supplementary Figure 31: ^{1}H and ^{13}C spectra of **HABI-O-*n*-oct**.

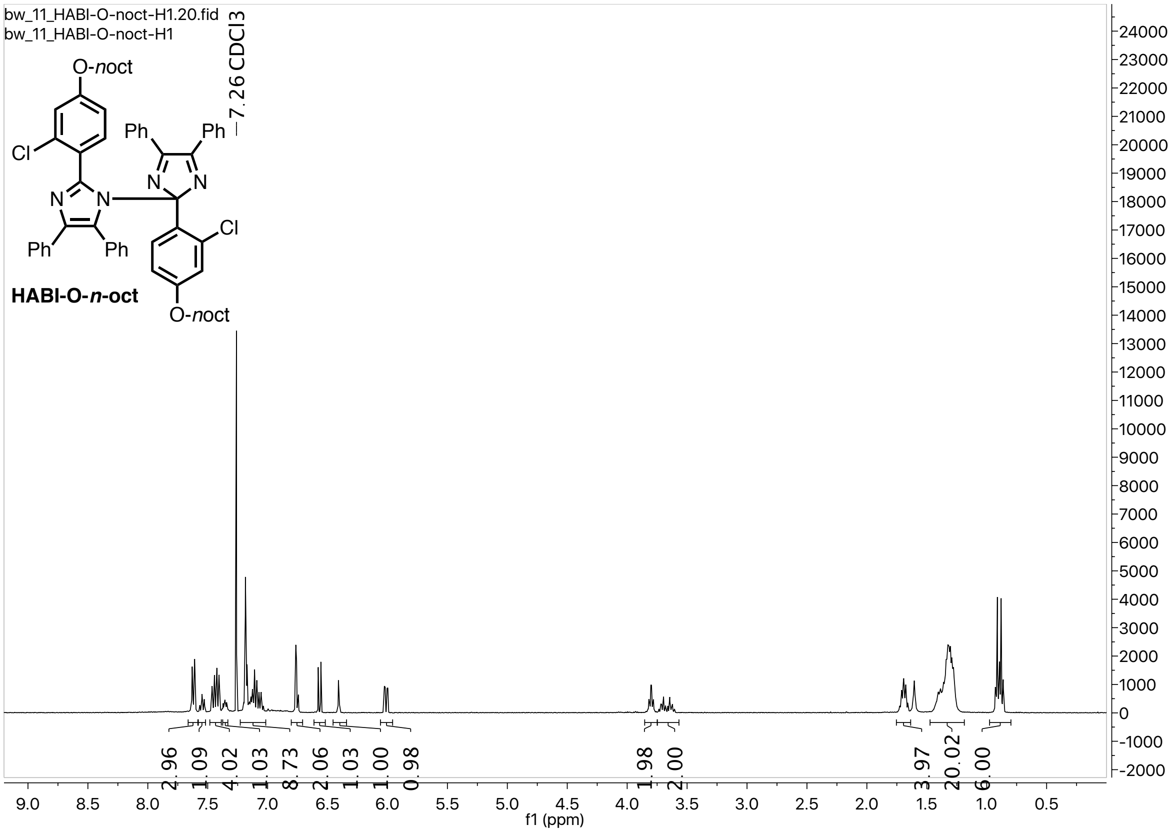

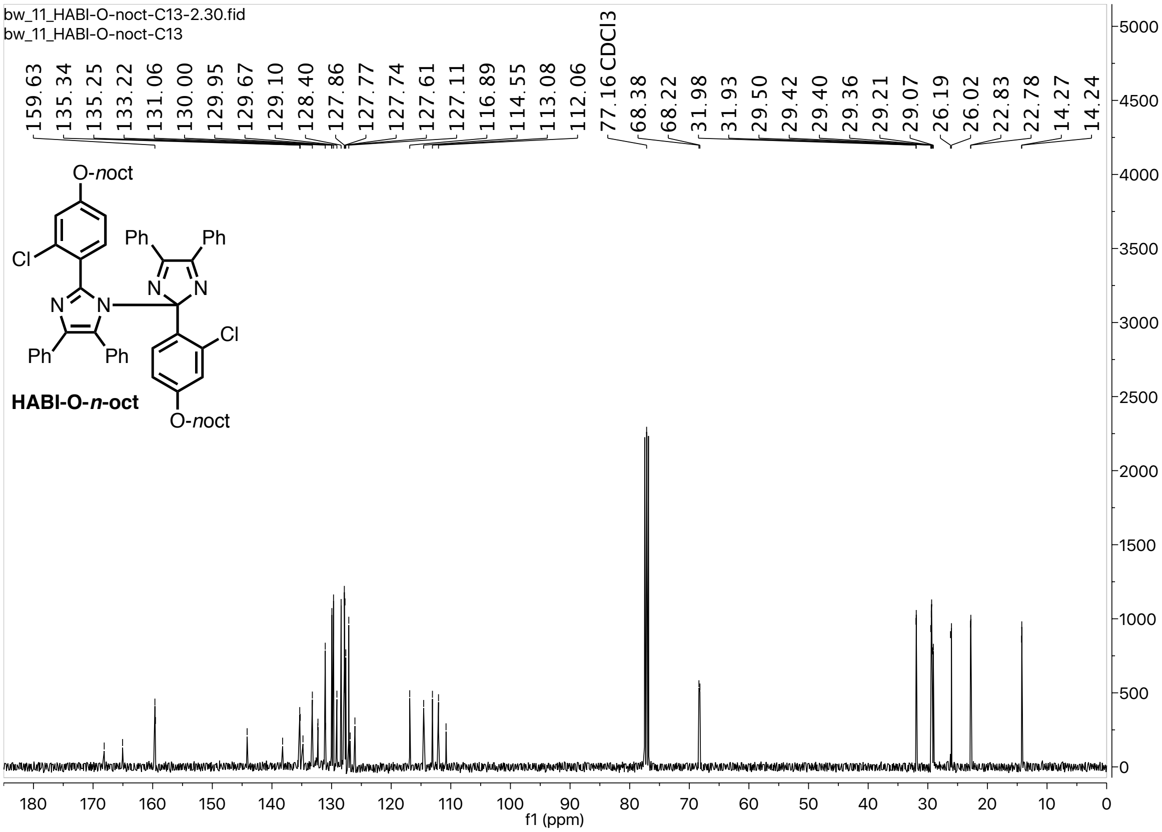

Supplementary Figure 32: ^{1}H and ^{13}C spectra of **PA-OH**.

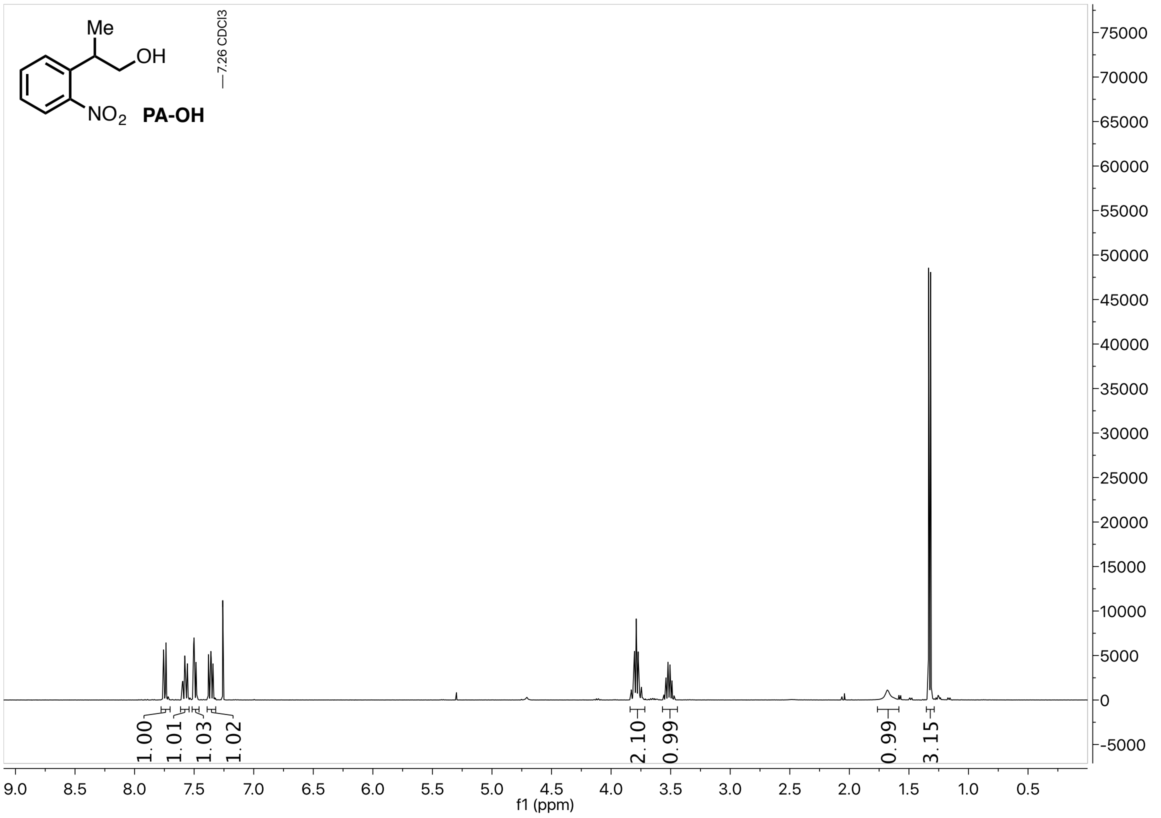

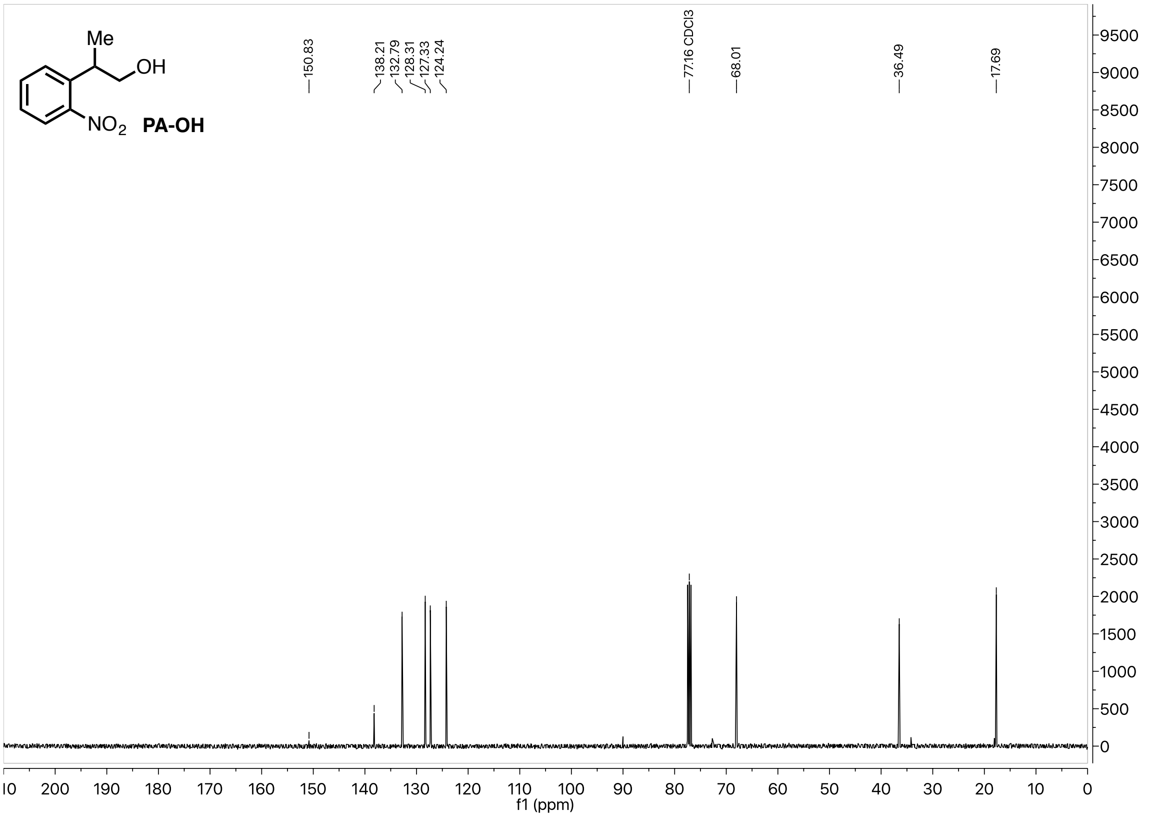

Supplementary Figure 33: ^{1}H and ^{13}C spectra of **PA**.

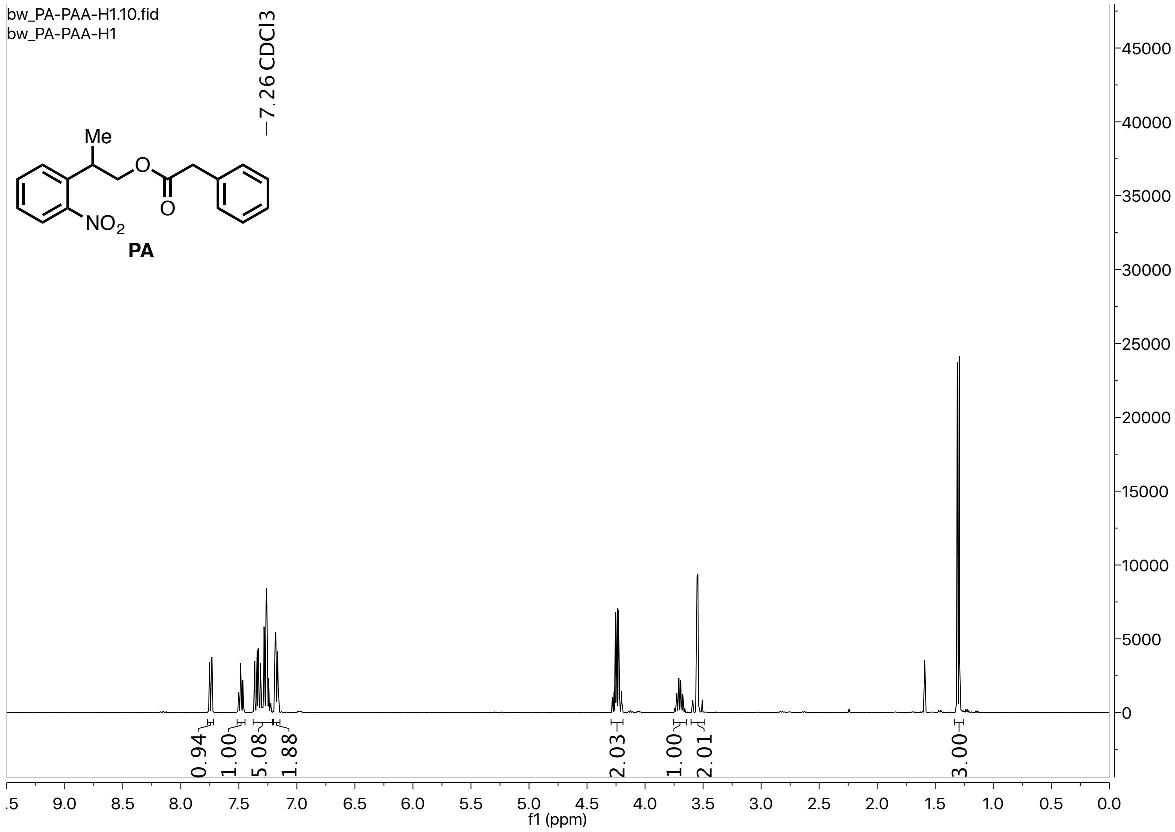

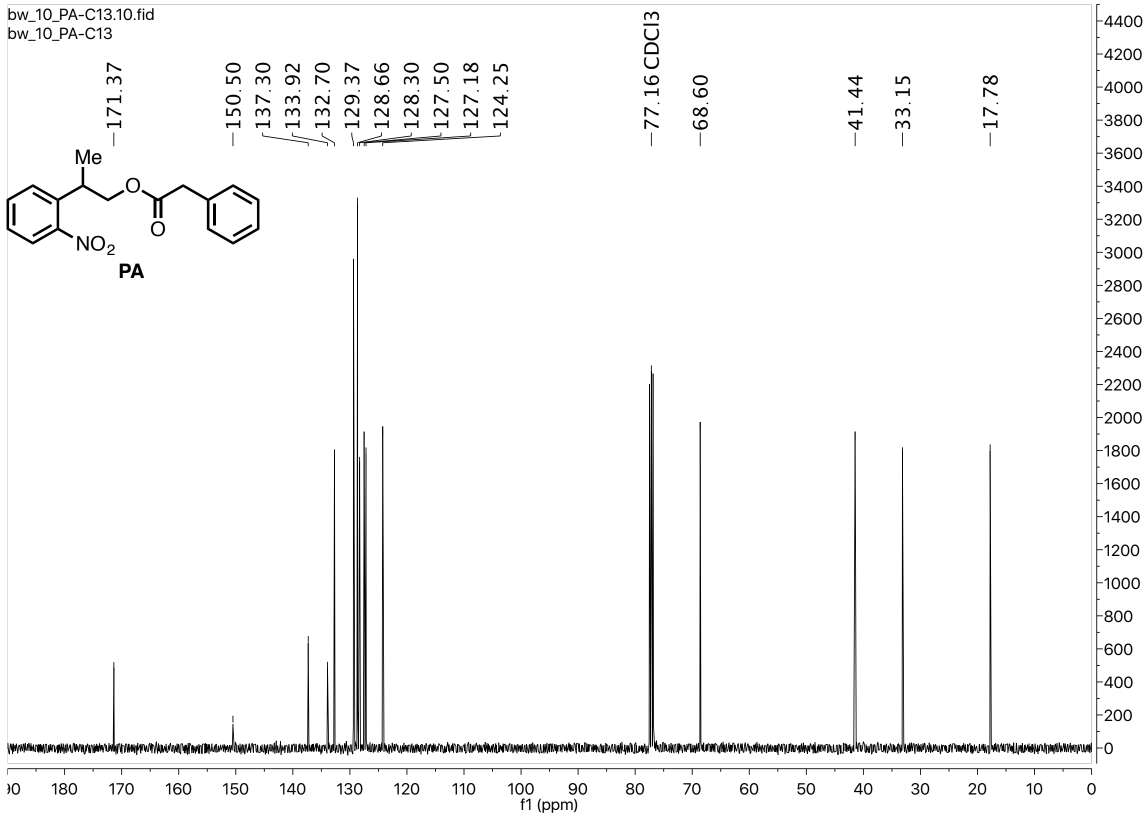

Supplement: Supplementary file 1 — Supplementary Information [file 41467_2018_5300_MOESM1_ESM.pdf]
